# The chemical reprogramming of unipotent adult germ cells towards authentic pluripotency and *de novo* establishment of imprinting

**DOI:** 10.1093/procel/pwac044

**Published:** 2022-11-17

**Authors:** Yuhan Chen, Jiansen Lu, Yanwen Xu, Yaping Huang, Dazhuang Wang, Peiling Liang, Shaofang Ren, Xuesong Hu, Yewen Qin, Wei Ke, Ralf Jauch, Andrew Paul Hutchins, Mei Wang, Fuchou Tang, Xiao-Yang Zhao

**Affiliations:** State Key Laboratory of Organ Failure Research, Department of Developmental Biology, School of Basic Medical Sciences, Southern Medical University, Guangzhou 510515, China; Beijing Advanced Innovation Center for Genomics, School of Life Sciences, Peking University, Beijing 100871, China; Biomedical Pioneering Innovation Center, Ministry of Education Key Laboratory of Cell Proliferation and Differentiation, Beijing 100871, China; State Key Laboratory of Organ Failure Research, Department of Developmental Biology, School of Basic Medical Sciences, Southern Medical University, Guangzhou 510515, China; Department of Plastic Surgery, Affiliated Hangzhou First People’s Hospital, Zhejiang University School of Medicine, Hangzhou 310006, China; State Key Laboratory of Organ Failure Research, Department of Developmental Biology, School of Basic Medical Sciences, Southern Medical University, Guangzhou 510515, China; State Key Laboratory of Organ Failure Research, Department of Developmental Biology, School of Basic Medical Sciences, Southern Medical University, Guangzhou 510515, China; State Key Laboratory of Organ Failure Research, Department of Developmental Biology, School of Basic Medical Sciences, Southern Medical University, Guangzhou 510515, China; State Key Laboratory of Organ Failure Research, Department of Developmental Biology, School of Basic Medical Sciences, Southern Medical University, Guangzhou 510515, China; State Key Laboratory of Organ Failure Research, Department of Developmental Biology, School of Basic Medical Sciences, Southern Medical University, Guangzhou 510515, China; State Key Laboratory of Organ Failure Research, Department of Developmental Biology, School of Basic Medical Sciences, Southern Medical University, Guangzhou 510515, China; Department of Urology, Nanfang Hospital, Southern Medical University, Guangzhou 510515, China; School of Biomedical Sciences, Li Ka Shing Faculty of Medicine, The University of Hong Kong, Hong Kong SAR, China; Department of Biology, Southern University of Science and Technology, Shenzhen 518055, China; State Key Laboratory of Organ Failure Research, Department of Developmental Biology, School of Basic Medical Sciences, Southern Medical University, Guangzhou 510515, China; Department of Neonatology, Zhujiang Hospital, Southern Medical University, Guangzhou 510280, China; Beijing Advanced Innovation Center for Genomics, School of Life Sciences, Peking University, Beijing 100871, China; Biomedical Pioneering Innovation Center, Ministry of Education Key Laboratory of Cell Proliferation and Differentiation, Beijing 100871, China; State Key Laboratory of Organ Failure Research, Department of Developmental Biology, School of Basic Medical Sciences, Southern Medical University, Guangzhou 510515, China; Guangdong Provincial Key Laboratory of Construction and Detection in Tissue Engineering, Southern Medical University, Guangzhou 510515, China; Department of Gynecology, Zhujiang Hospital, Southern Medical University, Guangzhou 510280, China; Key Laboratory of Mental Health of the Ministry of Education, Guangzhou 510515, China; Bioland Laboratory (Guangzhou Regenerative Medicine and Health Guangdong Laboratory), Guangzhou 510005, China

**Keywords:** reprogramming, spermatogonial stem cell, tetraploid complementation, imprinting

## Abstract

Although somatic cells can be reprogrammed to pluripotent stem cells (PSCs) with pure chemicals, authentic pluripotency of chemically induced pluripotent stem cells (CiPSCs) has never been achieved through tetraploid complementation assay. Spontaneous reprogramming of spermatogonial stem cells (SSCs) was another non-transgenic way to obtain PSCs, but this process lacks mechanistic explanation. Here, we reconstructed the trajectory of mouse SSC reprogramming and developed a five-chemical combination, boosting the reprogramming efficiency by nearly 80- to 100-folds. More importantly, chemical induced germline-derived PSCs (5C-gPSCs), but not gPSCs and chemical induced pluripotent stem cells, had authentic pluripotency, as determined by tetraploid complementation. Mechanistically, SSCs traversed through an inverted pathway of *in vivo* germ cell development, exhibiting the expression signatures and DNA methylation dynamics from spermatogonia to primordial germ cells and further to epiblasts. Besides, SSC-specific imprinting control regions switched from biallelic methylated states to monoallelic methylated states by imprinting demethylation and then re-methylation on one of the two alleles in 5C-gPSCs, which was apparently distinct with the imprinting reprogramming *in vivo* as DNA methylation simultaneously occurred on both alleles. Our work sheds light on the unique regulatory network underpinning SSC reprogramming, providing insights to understand generic mechanisms for cell-fate decision and epigenetic-related disorders in regenerative medicine.

## Introduction

Reprogramming describes the process of reverting the cell fate of differentiated cells to a pluripotent state and provides therapeutic potential for regenerative medicine. For the last half-century, great success had been achieved by the development of somatic cell reprogramming by nuclear transfer (Gurdon and Byrne, 2003; Byrne et al., 2007), transcription factors overexpression (Takahashi and Yamanaka, 2006; Takahashi et al., 2007), or chemical induction (Hou et al., 2013; Zhao et al., 2015) of pluripotency. However, although many regulatory networks and biological processes have been investigated in reprogramming, and many novel methods have been developed based on these insights, incomplete and defective patterns of epigenetic reprogramming remain a common occurrence in reprogramming (Li et al., 2010; Samavarchi-Tehrani et al., 2010; Takikawa et al., 2013; Zhao et al., 2015, 2018; Matoba et al., 2018; Yagi et al., 2019). This suggests there are several unknown mechanisms that remain to be characterized. Amongst the many reprogramming strategies, CiPSCs was a breakthrough, as it avoided the use of oocytes or genetic manipulation, making cell fate transitions potentially easier to control in a cost-effective and nonimmunogenic way (Hou et al., 2013). However, tetraploid complementation, the gold standard test for pluripotency and developmental potency, had been confirmed in embryonic stem cells (ESCs) and induced pluripotent stem cells (iPSCs) but not in CiPSCs (Zhao et al., 2009). This hints at functional deficiencies, likely epigenetic, in CiPSCs that compromises their full pluripotency capability.

Spermatogonial stem cells (SSCs) locate in the testis and initiate continuous spermatogenesis, these SSCs can be well maintained *in vitro* for several passages (Kanatsu-Shinohara et al., 2003). Intriguingly, in culture, a tiny majority of SSCs spontaneously convert to germline-derived pluripotent stem cells (gPSCs), in a form of non-transgenic reprogramming (Kanatsu-Shinohara et al., 2004; Guan et al., 2006; Ko et al., 2009). Interestingly, the spontaneous way of SSCs reprogramming is quite different from other cell fate transitions triggered by extra induction, suggesting distinct regulatory mechanisms in this biological process. However, the extremely low efficiency (about 0.05%) of spontaneous SSC reprogramming hinders the investigation of this unique phenomenon and the underlying mechanisms (An et al., 2017; Jeong et al., 2017), thus an efficient SSC to gPSC reprogramming system is urgently required. More importantly, the fully pluripotency of gPSCs had not been verified by the most stringent test for pluripotency, which stimulated us to improve the developmental potency of gPSCs.

In this study, we established a highly efficient (4.2%) SSC reprogramming system that can generate gPSCs via chemical induction. These gPSCs can form a complete mouse in tetraploid complementation assays, confirming their true pluripotency. Further, we showed that SSC reprogramming traversed through an inverted route of *in vivo* germ cell development and the chemical-induced gPSCs had correct imprinting status using single-cell multi-omics (transcriptome and DNA methylome) analysis. Our work shows the sophisticated regulatory network in cell fate transitions and the critical importance of the accurate re-establishment of imprinted pattern during reprogramming.

## Results

### Reconstruction of SSC reprogramming roadmap by single-cell RNA sequencing

**Figure 1.**
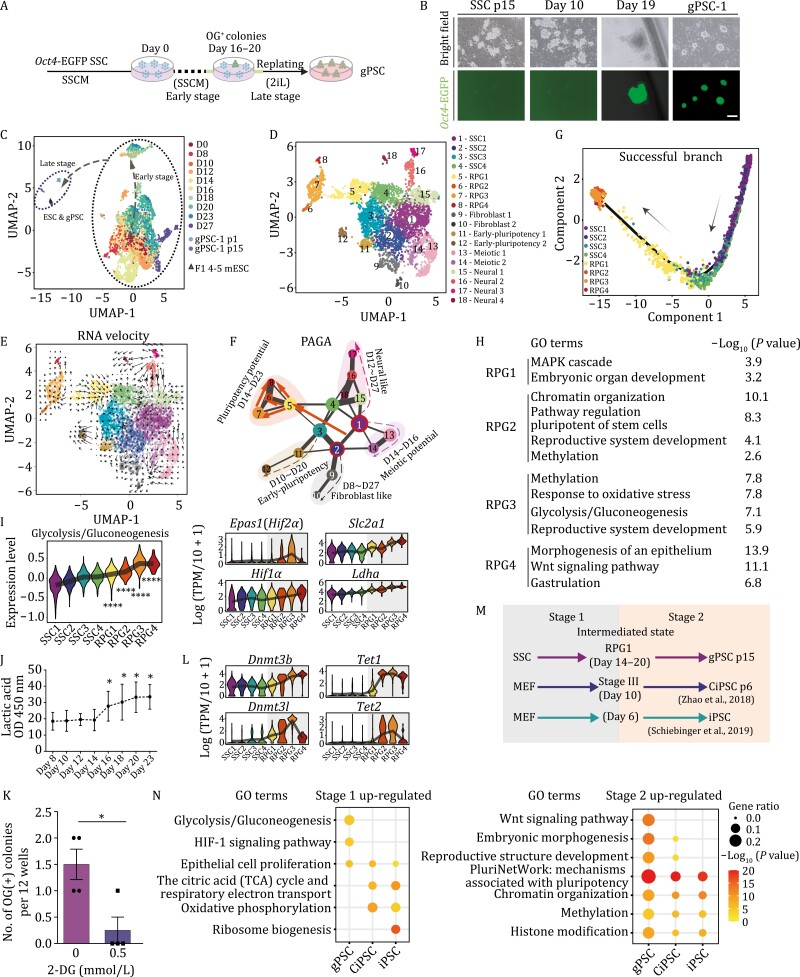
Single-cell RNA-sequencing roadmap of SSC reprogramming. (A) Schematic for the reprogramming of SSC. Days 16–20, *Oct4*-EGFP positive (OG(+)) colonies appeared. SSCM, spermatogonial stem cell culture medium. (B) Bright field (top) and fluorescence images (bottom) of *Oct4*-EGFP SSCs (days 0 and 10), *Oct4*-EGFP positive (OG(+)) colonies in the early stage of reprogramming (day 19), and gPSC-1 (p1) in the late stage of reprogramming. Scale bar, 200 μm. (C) Uniform manifold approximation and projection (UMAP) plot showing the transcriptome data of reprogramming cells, gPSCs, and ESCs. Reprogramming cells are colored by the day of reprogramming. (D) UMAP plot showing the transcriptome data of reprogramming cells from day 0 to day 27, cells are colored by clusters identified from graphed-based unsupervised clustering. (E) UMAP plot showing the RNA velocity vector field of the early stage of SSC reprogramming. (F) PAGA analysis showing the trajectories of the early stage of SSC reprogramming, each circle represents an individual cluster, the size of circles indicates the number of cells in clusters. (G) Cell trajectory of the successful branch of SSC reprogramming, inferred by Moonocle2. Arrows indicate the developmental order of these cells. (H) Enriched GO terms and *P* values of differential expressed genes of RPG1/2/3/4 clusters. (I) Violin plots showing the expression level of KEGG pathway ‘Glycolysis/Gluconeogenesis’ (mmu00010) and representative glycolysis-related genes. Dunn’s non-parametric test was performed for pairwise multiple comparison of activity between adjacent clusters, *P* values were adjusted by Bonferroni method, ****P < 0.0001. (J) Extracellular lactic acid level plotted over time during SSC reprogramming. Unpaired two-tailed Student’s *s*-test, *P < 0.05. (K) Bar plot showing the number of OG(+) colonies on day 23 of SSC reprogramming treated with 2-DG at different concentration. Unpaired two-tailed Student’s *t*-test, *P < 0.05. (L) Violin plots showing the expression levels of DNA methylation related genes. (M) Schematic showing the stage division of gPSC-, CiPSC-, and iPSC-reprogramming. (N) Bubble plots showing GO terms and their representative genes upregulated in stage 1 (left) and stage 2 (right) of three reprogramming processes, corresponding to (M). GO terms and representative genes are indicated by colors. See also Figs. S1 and S2.

Functional SSCs were established from postnatal day (PND) 5.5 *Oct4*-EGFP mice (also known as OG2, expression of EGFP is under the control of *Oct4* promoter) (Szabó et al., 2002), feature genes expression (GFRa1, ZBTB16, DDX4, and OCT4) and spermatogenesis potency of SSCs were carefully investigated (Fig. S1A and S1B). Spontaneous SSC-reprogramming efficiency has been reported to vary across laboratories, hence we adopted a relatively efficient and reproducible system created by Ko et al. (2009). After 16–20 days of cultivation without passaging, *Oct4*-EGFP positive (OG(+)) colonies with typical ESC morphology appeared amongst the SSC colonies. gPSCs were then established by transferring the ESC-like colonies to ground state ESC culture medium (2iL) (Ying et al., 2008). Thus, reprogramming in SSC medium and ESC medium were designated as the early and late stage of SSC reprogramming, respectively (Fig. 1A and 1B). We confirmed that key pluripotent markers (OCT4, NANOG, and SSEA1) were activated, and the germ cell marker DDX4 was silenced in gPSCs (Fig. S1C). Chromosome integrity and differentiation potential of gPSCs were examined by karyotype- and teratoma formation assays, respectively (Fig. S1D and S1E). Furthermore, developmental potency was corroborated by both germline transmission and chimera formation (Fig. S1F–I). We confirmed that gPSCs derived from mouse SSCs possessed pluripotency comparable to ESCs.

To decipher the sophisticated regulatory networks during SSC reprogramming, 4,464 individual cells from two batches of SSC reprogramming at ten different time points (from day 0 to day 27), together with gPSCs, ESCs were collected for scRNA-seq analysis (modified STRT-seq) (Figs. 1C, S1J and S1K; Table S1) (Dong et al., 2018). On average, 8348 expressed genes and 267,298 UMIs (unique molecular identifiers) were detected in each cell after quality control (Fig. S1L). We first performed UMAP (uniform manifold approximation and projection) and graph-based clustering on single cells in the SSC medium, and 18 clusters were detected (Figs. 1D, S1K and S1M). Expression of well-defined marker genes was used to identify the cell type or cell type bias within each cluster, including SSC-specific genes (*Zbtb16*, *Dazl*, and *Dmrt1*) (Schrans-Stassen et al., 2001; Costoya et al., 2004; Zhang et al., 2016), pluripotent genes (*Oct4*, *Nanog*, and *Sox2*) (Zhou et al., 2016), fibroblast specific genes (*Col12a1*, *Col5a2*, and *Ccn2*) (Schiebinger et al., 2019), meiosis-related genes (*Spo11* and *Dpep3*) (Romanienko and Camerini-Otero, 2000; Xie et al., 2019), early-pluripotent genes (*Esrrb*, *Dppa2*, and low level of *Oct4*) (Zhao et al., 2018), and neuro-related genes (*Nr2f1*, *Foxp2*, *Pitx2*, and *Msx1*) (Schiebinger et al., 2019). Based on these genes, we designated the 18 clusters as SSC1-4, reprogramming 1-4 (RPG1-4), fibroblast 1-2, early-pluripotent 1-2, meiotic 1-2, and neural 1-4 (Figs. 1D and S1N).

We next used RNA velocity, Monocole2, and partition-based graph abstraction (PAGA) analysis, to reconstruct the developmental trajectories (Figs. 1E, 1F, and S2A–C) (Trapnell et al., 2014; La Manno et al., 2018; Wolf et al., 2019). Briefly, a subset of SSCs acquired fibroblast identity (clusters 9 and 10); from day 10 to day 20, early-pluripotent features were gained in a group of SSCs (clusters 11 and 12); simultaneously, SSCs with neural characteristics appeared from day 12 to day 27 (clusters 15 to 18); some other SSCs could transiently express meiosis related genes from day 14 to day 16 (clusters 13 and 14); notably, a group of cells diminished SSC signatures and obtained pluripotent characters from day 14 to day 23 (clusters 5 to 8) (Figs. 1F and S2D).

### Hallmarks of the roadmap of SSC reprogramming

Among these cell-fate transition trajectories, we designated the route with pluripotent signatures (SSC1-4 and RPG1-4) as the successful reprogramming branch, whilst all other routes were classified as failed branches (Figs. 1G and S2E). Gene ontology (GO) analysis was performed on the differentially expressed genes (DEGs) in either successful- or failed reprogramming branches, respectively. ‘Embryo development’, ‘pluripotent stem cell’ as well as ‘chromatin modification’ related GO terms were enriched in the successful reprogramming branch; meanwhile, ‘extracellular matrix organization’, ‘meiotic division’ and ‘neural differentiation’ related events were occurred in the failed reprogramming branches (Figs. 1H and S2F; Table S2).

Intriguingly, glycolysis-metabolism-related terms (‘glycolysis/gluconeogenesis’) were enriched in the successful branch, and glycolysis related genes (including *Hif1a*, *Epas1* (*Hif2a*), *Slc2a1*, *Ldha*) were upregulated from SSC1 to RPG4, suggesting the activation of glycolysis during SSC reprogramming (Fig. 1H and 1I). Indeed, lactic acid was elevated after day 16 (Fig. 1J), and addition of 2-deoxy-d-glucose (2-DG, a glycolysis inhibitor) (Kanatsu-Shinohara et al., 2016), suppressed the emergence of gPSCs as measured by OG(+) colonies (Fig. 1K); importantly, glycolysis activation was mainly observed in the successful branch but not in the failed branches (Fig. S2G), implying a vital correlation between glycolysis and successful reprogramming. Recent studies have identified an intimate link between metabolic processes and epigenetic control, as many metabolic processes provide and control reaction substrates for epigenetic modifications in cell fate transitions (Ly et al., 2020; Tarazona and Pourquie, 2020). In our study, epigenetic regulation related GO terms (‘methylation’) and genes (*Dnmt3b*, *Dnmt3l*, *Tet1*, *Tet2*) were enriched in the successful branch of SSC reprogramming (Fig. 1H and 1L; Table S2). Moreover, the expression of both glycolysis- and DNA methylation-related genes was positively correlated with the pluripotent features from RPG1 to RPG4, suggesting the direct or indirect regulation of glycolytic-metabolism and DNA methylation/demethylation in SSC reprogramming (Fig. S2H; Table S2).

This comprehensive roadmap of SSC reprogramming to gPSCs will allow us to explore the unique and common events between SSC reprogramming and other reprogramming processes, including CiPSC and iPSC (Fig. 1M, see Methods) (Zhao et al., 2018; Schiebinger et al., 2019). Though few genes simultaneously activated or silenced among these three reprogramming processes, ‘epithelial cell proliferation’ and ‘pluripotency network’ were identified as the common events in all three reprogramming processes. Of note, the enrichment of glycolysis-related genes in the first stage of SSC reprogramming was apparently distinct with the relatively activation of ‘oxidative phosphorylation’ and ‘TCA cycle’ in MEF reprogramming (Figs. 1N and S2I; Table S3). Particularly, the downregulation of ‘spermatogenesis’- and ‘DNA methylation involved in gamete generation’-related genes were only observed in SSC reprogramming, which was due to the different origin of initial cells (Fig. S2J; Table S3).

### A high-efficient SSC reprogramming system developed by a five-chemicals combination

**Figure 2.**
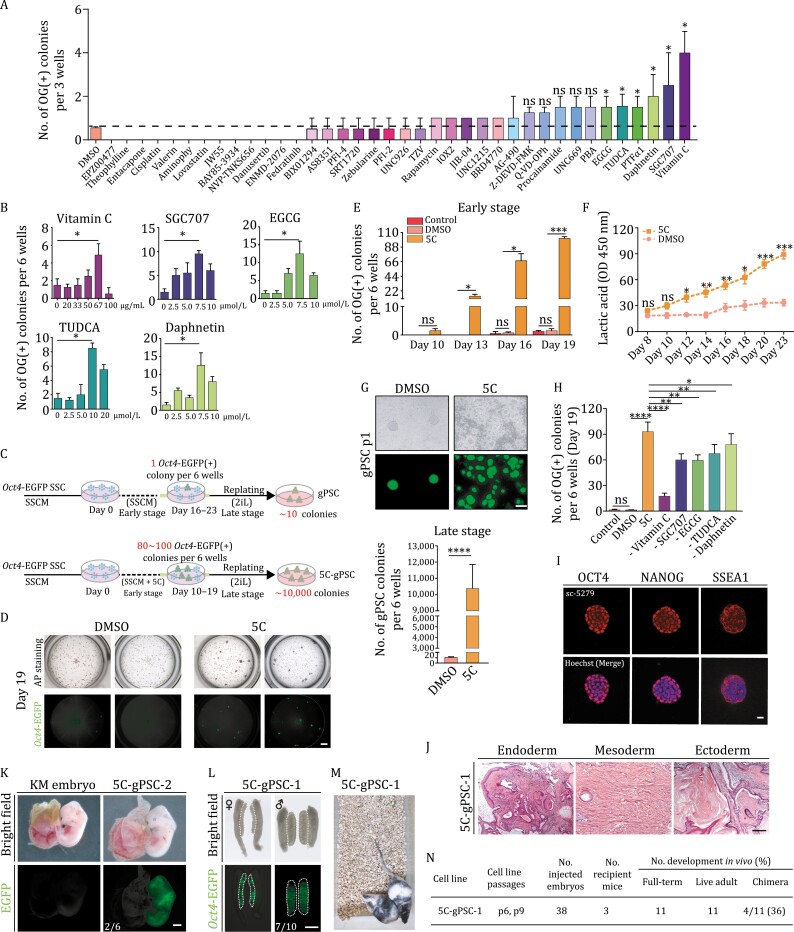
A robust SSC reprogramming system induced by small-molecule compounds. (A) Bar plots showing the number of OG(+) colonies on day 19 treated with individual chemical at 10 µmol/L or 50 μg/mL during SSC reprogramming. Unpaired two-tailed Student’s *s*-test was performed between DMSO and each chemical group, *P < 0.05. ns, not significant. (B) Bar plots showing the numbers of OG(+) colonies on day 19 treated with individual chemical at different concentration. Unpaired two-tailed Student’s *t*-test, *P < 0.05. (C) Schematic of SSC reprogramming with the original (top) and 5C (bottom) treatments. (D) AP staining (top) and fluorescence images (bottom) showing the reprogramming cells on day 19 in DMSO and 5C groups. Scale bar, 50 μm. AP staining, alkaline phosphatase staining. (E) Bar plot showing the number of OG(+) colonies at different time points in DMSO and 5C groups. (F) Line chart showing the extracellular lactic acid level plotted over time in DMSO and 5C groups. Unpaired two-tailed Student’s *t*-test, *P < 0.05, **P < 0.01, ***P < 0.001, ns, not significant. (G) Bright field and fluorescence images (top) of gPSCs (p1) from DMSO and 5C groups, respectively. Bar plot (bottom) showing the numbers of gPSC colonies (p1) from these 2 groups. Unpaired two-tailed Student’s *t*-test; ****P < 0.0001. (H) Bar plot showing the numbers of OG(+) colonies on day 19 in the indicated groups, the original condition (control), DMSO, 5C and 5C withdrawing different chemicals. Unpaired two-tailed Student’s *t*-test, *P < 0.05, **P < 0.01, ****P < 0.0001, ns, not significant. (I) Immunofluorescence of OCT4, NANOG and SSEA1 (red) in 5C-gPSC-1. Scale bar, 20 μm. (J) H&E staining of paraffin sections of teratoma derived from subcutaneous injection of 5C-gPSC-1 into nude mice. Scale bar, 100 μm. (K) Bright field and fluorescence images showing E12.5 embryo of KM mouse and chimaera embryo produced by the diploid blastocyst injection of CAG-EGFP 5C-gPSC-2, 2 out of 6 embryos were chimaeras after blastocyst injection. Scale bar, 2 mm. (L) Bright filed and fluorescence images of the E12.5 genital ridges derived from diploid blastocyst injection of 5C-gPSC-1, 7 out of 10 pairs of genital ridges were GFP positive after blastocyst injection. Scale bar, 500 μm. (M) Chimaeras produced from B6D2F1 (black coat color) 5C-gPSC-1. (N) Summary of chimeric assays of diploid blastocyst injection using 5C-gPSC-1.

In order to improve the SSC reprogramming system, we carried out small-molecule screening by adding 185 candidate small molecules chosen according to our single cell analysis as well as previous studies (Table S4) (Takashima et al., 2013). Interestingly, SGC707 (a PRMT3 inhibitor), vitamin C (a DNA demethylation related chemical), (–)-Epigallocatechin Gallate (EGCG, a natural product regulating DNA methyltransferase), Daphnetin (a natural coumarin derivatives inhibiting protein kinase), PTFα1 (a p53 inhibitor), and tauroursodeoxycholic acid (TUDCA, an anti-apoptosis chemical) were identified to significantly improve the efficiency of SSC reprogramming, respectively (Fig. 2A and 2B) (Takashima et al., 2013). To preserve the genomic integrity of gPSCs, PTFα1 was excluded in the subsequent studies. We then asked if the combination of all these five chemicals, SGC707, vitamin C, EGCG, Daphnetin, and TUDCA (named 5C hereafter) could synergistically improve the efficiency of SSC reprogramming. Notably, in the early stage of reprogramming, approximately 80–100 times more OG(+) colonies as well as significant elevation of lactic acid were observed in 5C treatment than in the control (Fig. 2C–F). Of note, 5C treatment not only significantly improved reprogramming efficiency, but also accelerated the reprogramming progress, as OG(+) and ESC-like colonies could be detected as early as day 10 (Fig. 2E). Moreover, the number of gPSC colonies with 5C treatment was nearly 1000 times more than the control after replating to ESC medium in the late stage of reprogramming (Fig. 2C and 2G).

We next evaluated if each chemical in 5C cocktail was necessary or sufficient for the high efficiency of gPSC reprogramming, by withdrawal experiments. The results indicated that all those chemicals contributed to increasing the efficiency of reprogramming, and vitamin C was a dominant one but it could not substitute the other four chemicals in 5C (Fig. 2H and 2B). Finally, we confirmed the pluripotency of gPSCs derived from 5C treatment (5C-gPSCs) by marker genes expression, teratoma formation, germline transmission, and chimera formation (Fig. 2I–N). Thus, 5C-gPSCs are functionally comparable to ESCs.

### 5C-gPSCs displayed superior developmental potency and less growth deficiency than gPSCs, as determined by tetraploid complementation

**Figure 3.**
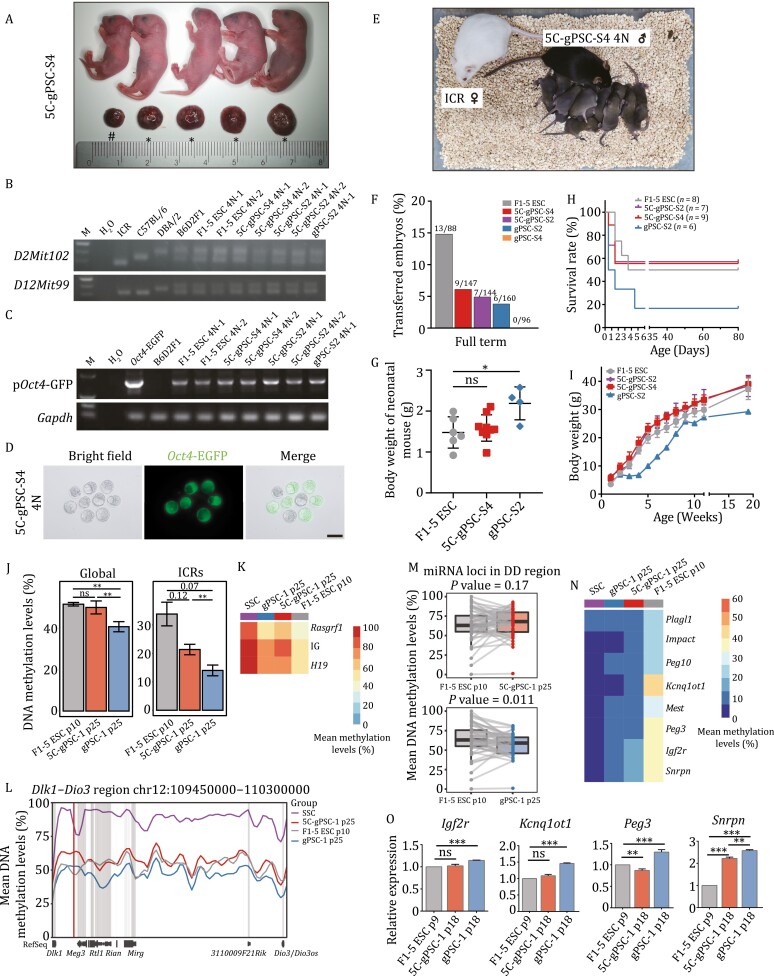
5C-gPSCs had true pluripotency identified by tetraploid complementation assay. (A) 5C-gPSC 4N-comp pups and their corresponding placentae. Note: * Pups survived beyond 4 days postnatal; # Pup died within 4 days postnatal. (B) Simple sequence length polymorphism (SSLP) analysis for lineage identification covers markers from different chromosomes, and the 4N-comp mice showed a polymorphic pattern similar with that from the F1-5 ESC, 5C-gPSC-S4, or gPSC-S2 cells originating from B6D2F1 mice, and different from the ICR, C57, or DBA mice. M denotes molecular mass marker. (C) Genotyping analysis of *Oct4*-EGFP from F1-5 ESC, 5C-gPSC-S4, or gPSC-S2 4N-comp mice. (D) Bright field (left), fluorescence (middle) and merged (right) images of E3.5 *Oct4*-EGFP blastocysts flushed from an ICR female mouse mated with an 5C-gPSC-S4 4N-comp mouse. Scale bar, 100 μm. (E) An 11-week-old 5C-gPSC-S4 4N-comp mouse with a uniformly black coat and its F_1_ progeny from its mating to an ICR female mouse. (F) Bar plot showing the developmental rate of full-term 4N-comp pups derived from PSCs, numbers of animals obtained per total number of transferred embryos are shown. Groups are indicated by colors. See Table 1 for details. (G) Dot plot showing the body weight of 4N-comp mice born by Caesarean section in this study on day 0 postnatal. Groups are indicated by colors. Unpaired two-tailed Student’s *t*-test, *P < 0.05, ns, not significant. (H) Survival curves of 4N-comp mice obtained in this study. n, total numbers of 4N-comp mice derived from each group. Groups are indicated by colors. (I) Growth curves of 4N-comp mice obtained in this study at the indicated weeks postnatal. Groups are indicated by colors. (J) Bar plot showing the DNA methylation level of the whole genome (left) and total ICRs (right) in ESCs (p10), 5C-gPSCs (p25), gPSCs (p25), respectively. Unpaired two-tailed Student’s *t*-test, *P* values were shown above the lines, **P < 0.01, ns, not significant. (K) Heatmaps showing the mean methylation levels of the paternal ICRs in SSCs (p20), gPSCs (p25), 5C-gPSCs (p25), ESCs (p10). The color key from dark blue to red indicates low to high levels, respectively. (L) Line plot shows the mean methylation levels of *Dlk1-Dio3* region in SSCs (p20), 5C-gPSCs (p25), ESCs (p10), gPSCs (p25), respectively. Grey shadows indicate gene regions, the red shadow indicates IG-*Gtl2* ICR region. (M) Box plots show the mean DNA methylation levels across gene bodies (from transcription start site (TSS) to transcription end site (TES)) and the 50-bp flanking regions of each miRNA encoded locus in *Dlk1-Dio3* region in ESCs, gPSCs and 5C-gPSCs. One dot represents an miRNA encoded locus, the same loci in distinct PSCs are connected by grey lines. Paired two-tailed Student’s *t*-test was performed between ESCs and other PSCs. (N) Heatmaps showing the mean methylation levels of the maternal ICRs in SSCs (p20), gPSCs (p25), 5C-gPSCs (p25), ESCs (p10). The color key from dark blue to red indicates low to high levels, respectively. (O) qPCR analysis of relative expression of *Igf2r*, *Kcnq1ot1*, *Peg3*, and *Snrpn* normalized to the geometric mean of *Rps2* and *Gapdh* in ESCs, gPSCs, and 5C-gPSCs. Unpaired two-tailed Student’s *t*-test, **P < 0.01, ***P < 0.001, ns, not significant. See also Fig. S3.

Besides the efficiency improvement, we also wondered whether the chemical treatment was beneficial to the developmental potency of gPSCs. To this end we performed tetraploid complementation assay (‘gPSC 4N-comp’). When *Oct4*-EGFP PSCs were injected into tetraploid ICR blastocysts, normal E12.5 fetus and live pups could be developed from 5C-gPSCs, gPSCs, and ESCs (Figs. 3A, S3A, and S3B; Table 1). Simple sequence length polymorphism (SSLP) and transgenic analyses followed by PCR confirmed the lineage of the tetraploid complementation mice (Fig. 3B and 3C; Table S4). Additionally, all of the *Oct4*-EGFP 4N-comp mice were black B6D2F1 males; when mated with white ICR females, gPSC, 5C-gPSC and ESC 4N-comp mice could produce OG(+) blastocysts with normal morphology and offspring with a uniform brown coat color (Figs. 3D, 3E, and S3C–E). Collectively, these data demonstrated the developmental pluripotency of 5C-gPSCs and gPSCs to produce 4N-comp mice.

**Table 1.** Development of tetraploid complementation.

Cell line	No. of passages	No. of transferred embryos (A + B)	No. of normal embryos at E12.5 (%A)	No. of implantation site (%B)	No. of embryos arrested 10.5–18.5 (%B)	No. of full-term pups (%B)	No. of pups died within 4 days postnatal (%FT)	No. of pups surviving beyond 4 days (%FT)
F1-5 ESC	p7, p11	88	—	4 (4.5)	40 (45.5)	13 (14.8)	5 (38.5)	8 (61.5)
		(0 + 88)						
F1-6 ESC	p6	31	6 (19.4)	—	—	—	—	—
		(31 + 0)						
5C-gPSC-S4	p7, p15	179	1 (3.1)	50 (34.0)	69 (46.9)	9 (6.1)	4 (44.4)	5 (55.6)
		(32 + 147)						
5C-gPSC-S2	p14	192	4 (8.3)	4 (2.8)	46 (31.9)	7 (4.9)	3 (42.9)	4 (57.1)
		(48 + 144)						
gPSC-S2	p10, p11, p14, p17	330	3 (1.8)	42 (26.3)	57 (35.6)	6 (3.8)	5 (83.3)	1 (16.7)
		(170 + 160)						
gPSC-S3	p10	112	0	13 (81.3)	3 (18.7)	0	0	0
		(96 + 16)						
gPSC-S4	p15, p20	96	—	30 (31.3)	12 (12.5)	0	0	0
		(0 + 96)						
R-1 CiPSC	p7, p17, p22, p24	240	3 (18.8)	89 (39.7)	59 (26.3)	0	0	0
		(16 + 224)						
K-2 CiPSC	p6, p8, p9, p11, p14, p16	364 (115 + 249)	0	73 (29.3)	80 (32.1)	0	0	0
D-1 CiPSC	p3, p7	149	—	48 (32.2)	40 (26.8)	0	0	0
		(0 + 149)						

A, E12.5; B, E19.5; FT, full term.

Though gPSCs and 5C-gPSCs could both support intact embryo development of 4N-comp mice, the chance to get viable offspring from gPSCs was lower than that of 5C-gPSCs (0% and 3.8% vs. 4.9% and 6.1%; Fig. 3F, Table 1). Furthermore, 4N-comp mice derived from gPSCs showed heavier neonatal body weight, much lower survival rate and growth curve than that from 5C-gPSCs and ESCs (Fig. 3G–I; Table 1). These deficiencies suggested that gPSC 4N-comp mice harbored some epigenetic disorders, as previously reported (Choi et al., 2017; Li et al., 2017; Yagi et al., 2017). DNA methylation analysis by bisulfite-sequencing demonstrated that the proper methylation level of whole genome and total imprinting control regions (ICRs) were observed in ESCs and 5C-gPSCs, but not in gPSCs (Figs. 3J and S3F). Specifically, the paternal imprinting reprogrammed from SSCs to both gPSCs and 5C-gPSCs (Fig. 3K). Previously, it had been verified the closely correlation between the silencing of paternal imprinted *Dlk1-Dio3* region (DD region) as well as miRNAs in this cluster and the developmental potency of iPSCs (Liu et al., 2010; Stadtfeld et al., 2010). In our study, the DNA methylation of DD region and miRNAs encoded locus in this cluster preserved a relatively moderate level in ESCs and 5C-gPSCs; however, significant hypomethylation of DD regions and miRNA locus were observed in gPSCs (Fig. 3L and 3M). Besides, the methylation status of maternal imprinting control regions (ICRs) was partially reset in 5C-gPSCs, but not in gPSCs, meanwhile, specific maternal imprinted genes overexpression (*Igf2r*, *Kcnq1ot1*, *Peg3*, and *Snrpn*) were observed in gPSCs (Figs. 3N, 3O, and 7E). Together, these data suggested a positive-correlation between proper imprinting status of 5C-gPSCs and their subsequent developmental and growth advantages in tetraploid-derived mice.

**Figure 4.**
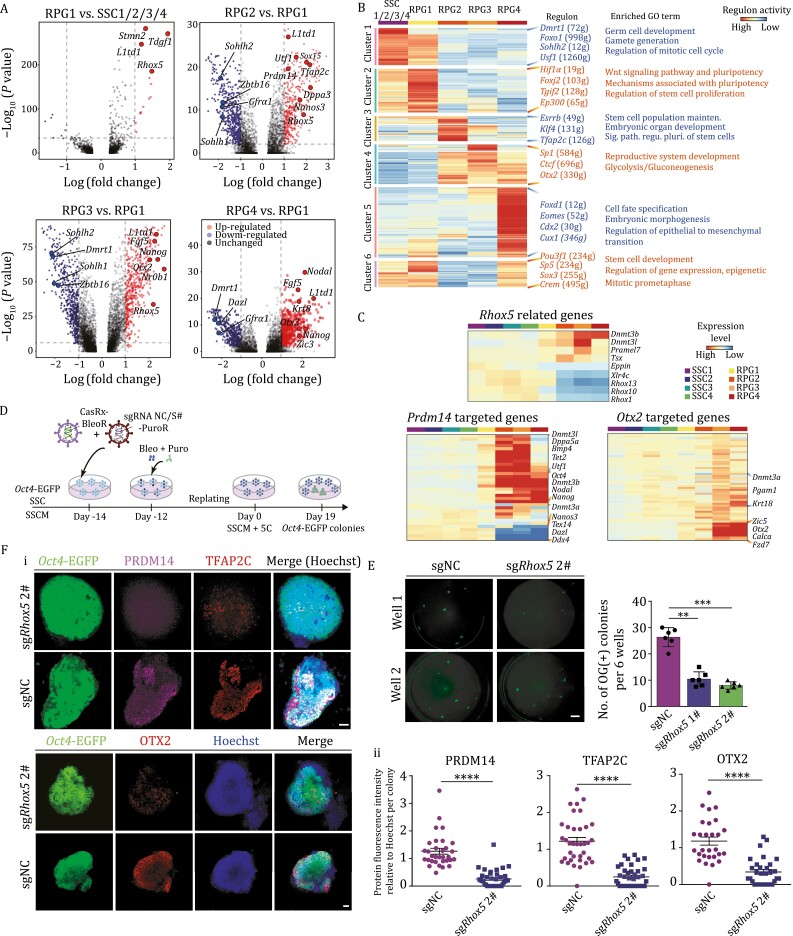
A reversed developmental trajectory of germ cell in SSC reprogramming. (A) UMAP plot showing the integration of single-cell transcriptome data from successful branch of SSC reprogramming (DMSO- and 5C groups), mouse *in vivo* germ cells and gonadal somatic cells from E6.5 to PND5.5. Grey dots indicate *in vivo* germ cells or germline somatic cells, colored dots or triangles with black outline indicate reprogramming cells or ESCs. (B) Scmap projection of the successful branch of SSC reprogramming (left, 5C group; right, DMSO group) and mouse *in vivo* germ cells. The color key from bright yellow to dark blue indicates low to high ratio of projected cells. SPG, spermatogonia; *in vivo* GC dev., *in vivo* germ cell development. (C) Heatmap showing the expression of DEGs of *in vivo* germ cell development and their expression dynamics in SSC reprogramming, GO terms enriched in both processes were highlighted. (D) Violin plots showing the expression levels of canonical marker genes of *in vivo* germ cells in the successful branch of SSC reprogramming. (E) Immunofluorescence of *Oct4*-EGFP (green) co-stained with RHOX5 (top, red) and L1TD1 (bottom, red), respectively, in domed colonies during SSC reprogramming. Scale bar, 50 μm. (F) Immunofluorescence of *Oct4*-EGFP (green) co-stained with PRDM14 (pink) and TFAP2C (red) in domed colonies during SSC reprogramming. Scale bar, 50 μm. (G) Immunofluorescence of *Oct4*-EGFP (green) co-stained with OTX2 (red) in domed colonies during SSC reprogramming. Scale bar, 50 μm. (H) Immunofluorescence of *Oct4*-EGFP (green) co-stained with PRDM14 (pink) and OTX2 (red) in domed colonies at different time points during SSC reprogramming. Note, * The diameters of detected colonies. Scale bar, 50 μm. (I) Boxplot showing the number of PRDM14(+)OTX2(–), PRDM14(+)OTX2(+), PRDM14(–)OTX2(+) colonies (left) and their percentage (right) in OG(+) colonies at different time points during SSC reprogramming, corresponding to Fig. 4H. See also Fig. S4.

**Figure 5.**
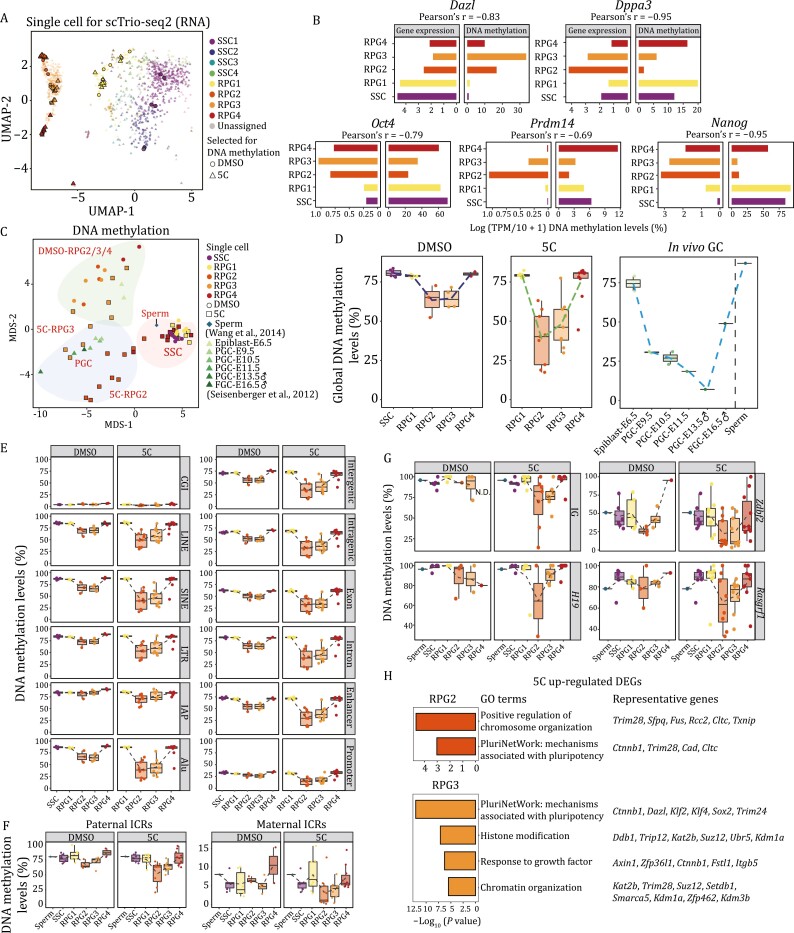
Identification of key regulators in SSC reprogramming. (A) Volcano plots showing differentially expressed genes (DEGs, | log (fold change) | ≥ 1, adjusted *P* < 0.01) in RPG1/2/3/4. Red and blue dots indicate upregulated and downregulated genes, respectively. Transcription factors are highlighted in bold. (B) Heatmap showing the regulon activities from SCENIC analysis in the successful branch of SSC reprogramming and representative enriched GO terms of indicated regulons. The color key from blue to red indicates low to high regulon activity. (C) Heatmaps showing the expression pattern of *Rhox5* related genes, target genes of *Prdm14* and *Otx2*, respectively. The color key from blue to red indicates low to high expression level. (D) Schematic of the gene editing by CRISPR-CasRx in SSC reprogramming. (E) Fluorescence images (left) and boxplot (right) showing the number of OG(+) colonies reprogrammed from empty vector (sgNC)–, vectors targeting *Rhox5* (sg*Rhox5* 1# and sg*Rhox5* 2#)-transfected SSCs in 5C condition on day 19. Scale bar, 50 μm. Unpaired two-tailed Student’s *t*-test, **P < 0.01, ***P < 0.001. NC, negative control. (F) (i) Immunofluorescence of *Oct4*-EGFP (green) co-stained with PRDM14 (top, pink), TFAP2C (top, red), and OTX2 (bottom, red) in domed colonies reprogrammed from sgNC- and sg*Rhox5* 2#-transfected SSCs, respectively. Scale bar, 50 μm. (ii) Dot plots showing the intensity of PRDM14, TFAP2C, and OTX2 corresponding to (i), Unpaired two-tailed Student’s *t*-test, ****P < 0.0001. See also Fig. S5.

**Figure 6.**
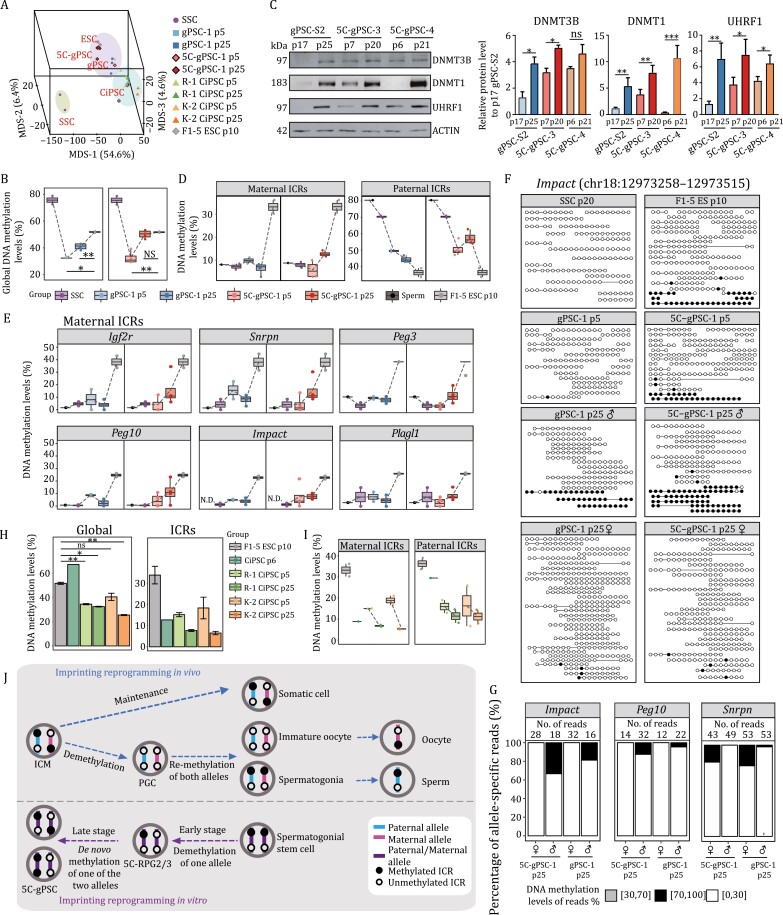
Single-cell methylation sequencing of the early stage of SSC reprogramming. (A) UMAP plot based on the transcriptome data of DMSO- or 5C-induced reprogramming cells. Cells are colored by clusters, colored dots or triangles with black outline indicate single cells selected for DNA methylation analysis. (B) Bar plots showing the expression and DNA methylation level of representative genes in SSC and RPG1/2/3/4, respectively. (C) Multidimensional scaling (MDS) analysis on the DNA methylome data of SSC reprogramming cells, SSC, mouse *in vivo* germ cells (Seisenberger et al., 2012) and sperm (Wang et al., 2014). Three background colors indicate distinct unsupervised clusters (see Fig. S6F). (D) Boxplots showing the global DNA methylation dynamics during DMSO (left) and 5C (middle) induced reprogramming processes and *in vivo* germ cells (right). Unpaired two-tailed Student’s *t*-test was performed in corresponding clusters of DMSO and 5C groups, respectively, *P < 0.05. (E) Boxplots showing the DNA methylation dynamics of indicated genomic features during DMSO and 5C induced reprogramming processes, respectively. (F) Boxplots showing the mean DNA methylation dynamics of paternal (left) and maternal (right) ICRs during DMSO and 5C induced reprogramming, respectively. The DNA methylation level of ICRs in sperm was the reference for other samples (Wang *et al*., 2014). (G) Boxplots showing the DNA methylation dynamics of representative paternal ICRs during DMSO and 5C induced reprogramming processes as well as sperm (Wang *et al*., 2014), respectively. (H) Enriched GO terms in the upregulated genes and representative genes of RPG2 and RPG3 from 5C group than that from control and DMSO groups. See also Fig. S6.

**Figure 7.**
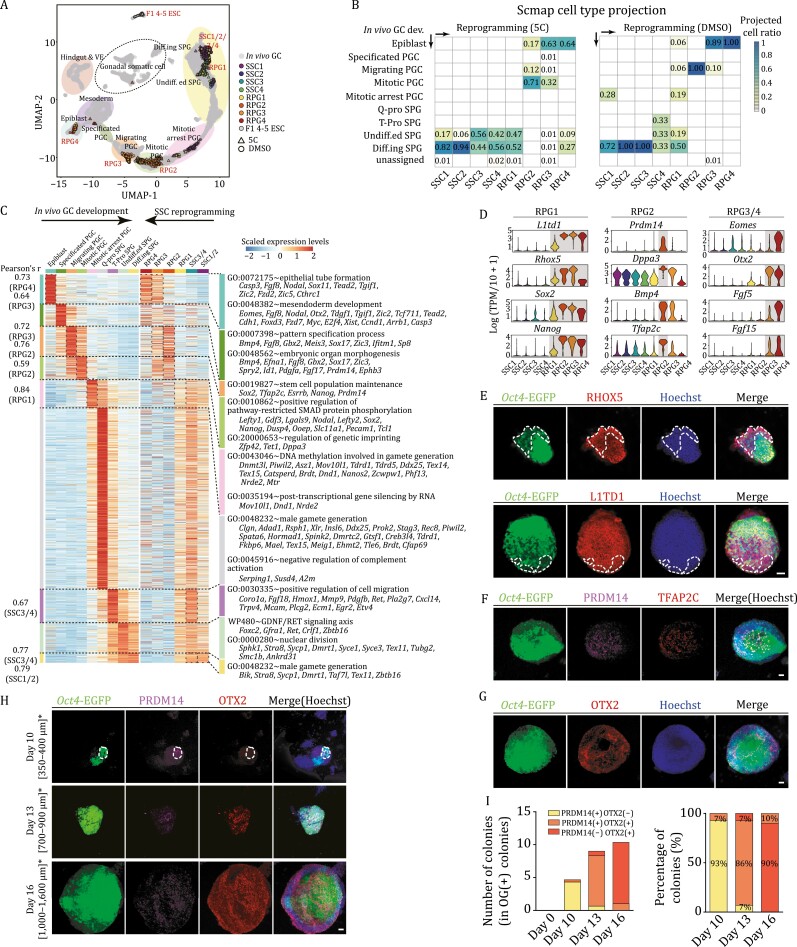
DNA methylation dynamics of the late stage of SSC reprogramming. (A) Three-dimensional MDS analysis on the DNA methylome (1-kb windows) of SSCs, ESCs, different passages of gPSCs, 5C-gPSCs and CiPSCs. Three background colors indicate distinct unsupervised clusters (see Fig. S7D). (B) Boxplots showing the global methylation dynamics of SSCs, ESCs, different passages of gPSCs and 5C-gPSCs. Unpaired two-tailed Student’s *t*-test, *P < 0.05, **P < 0.01, ns, not significant. (C) Western blot analysis of the protein levels of DNMT3B, DNMT1, and UHRF1 in different passages of gPSCs and 5C-gPSCs (left). Bar plot showing the grayscale intensity analysis of DNMT3B, DNMT1, and UHRF1, with ACTIN as the internal control (right). Unpaired two-tailed Student’s *t*-test, *P < 0.05, **P < 0.01, ***P < 0.001, ns, not significant. (D) Boxplots showing the DNA methylation level of maternal and paternal ICRs in sperm (Wang *et al*., 2014), SSCs, ESCs, different passages of gPSCs and 5C-gPSCs. (E) Boxplots showing the DNA methylation level of representative maternal ICRs in sperm (Wang *et al*., 2014), SSCs, ESCs, different passages of gPSCs and 5C-gPSCs. N.D., not detected. (F) Lollipop methylation diagrams showing the DNA methylation level of *Impact* ICR in gPSCs and 5C-gPSCs (p5 and p25) by sequencing reads in single CpG site resolution on paternal or maternal alleles. The DNA methylation level of ICRs in SSC and ESC was the reference for other samples. White and black dots indicate unmethylated and methylated CpG sites, respectively. (G) Proportion of DNA methylation levels of individual allele-specific sequencing read of ICRs (*Impact*, *Peg10*, and *Snrpn*) in p25 gPSCs and 5C-gPSCs. Number of covered reads for each allele was listed. (H) Bar plot showing the DNA methylation level of the whole genome (left) and total ICRs in CiPSCs (p6, Zhao *et al*., 2018), R-1 CiPSCs (p5 and p25), K-2 CiPSCs (p5 and p25), respectively. Unpaired two-tailed Student’s *t*-test, *P < 0.05, **P < 0.01, ns, not significant. (I) Boxplots showing the DNA methylation level of maternal and paternal ICRs in ESCs and different passages of CiPSCs. (J) Summary of the study. SSCs underwent imprinting demethylation in the early stage and *de novo* methylation of one of the two alleles in the late stage of 5C-induced SSC reprogramming. Biallelic methylation status of parental ICRs in SSCs converted to monoallelic methylation status in 5C-gPSCs through this stepwise spontaneous reprogramming. This imprinting reprogramming was apparently distinct with that of *in vivo* development, as ICRs demethylation from ICM to PGC and re-methylation of both alleles in spermatogonia and immature oocytes, and imprinting maintenance in somatic cell differentiation. ICR, imprinting control region; ICM, inner cell mass. See also Fig. S7.

### SSCs were reprogrammed to a pluripotent state along a reversed germline differentiation trajectory

To further decode the regulatory mechanism by which gPSCs achieved reprogramming, scRNA-seq was performed throughout the SSC reprogramming process induced by 5C and under control conditions (Fig. S4A–C; Table S1). Previously, the enrichment of GO terms related to ‘reproductive system development’ and ‘gastrulation’ in the successful branch suggested a correlation between SSC reprogramming and embryonic germ cell development (Fig. 1H). Thus, to integrate the analysis we included data from mouse male germ cells and somatic cells in the genital ridge or testis from embryonic day (E) 6.5 to postnatal day (PND) 5.5 (Zhao et al., 2021). Intriguingly, single cells from the successful reprogramming branch fitted much better with the *in vivo* germ cells, compared to gonadal somatic cells and embryonic stem cells after data integration (Figs. 4A, S4D, and S4E). In depth analysis suggested that regardless of the SSC reprogramming conditions, SSC1/2/3/4 resembled undifferentiated- and differentiating spermatogonia, and a subpopulation of RPG1 shared features with mitotic arrest primordial germ cell (mitotic arrest PGC); signatures of RPG2 were comparable to mitotic- and migrating PGC, and RPG3/4 acquired representative characteristics of the epiblasts (Figs. 4B and S4F) (Kiselev et al., 2018).

Furthermore, the upregulation of 836 genes out of 1425 stage-specific DEGs of *in vivo* germ cell development were captured in the corresponding clusters during SSC reprogramming, reflecting the acquisition of fetal germ cell (FGC) features and the loss of spermatogenesis signatures in this reprogramming process (Fig. 4C; Table S5). For instance, pluripotent markers *L1td1*, *Rhox5*, *Sox2*, and *Nanog* first expressed in RPG1; PGC marker genes *Prdm14*, *Dppa3*, *Bmp4*, and *Tfap2c* were predominantly expressed in RPG2; epiblast-related markers *Eomes*, *Otx2*, *Fgf5*, and *Fgf15* were exclusively expressed in RPG3/4 (Figs. 4D and S4G). Immunostaining verified the expression patterns of these germ cell markers (RHOX5, L1TD1, PRDM14, TFAP2C, DPPA3, and OTX2) during SSC reprogramming (Figs. 4E–G and S4H). In addition, the expression of RPG2 specific marker (PRDM14) and RPG3/4 marker (OTX2) in OG(+) colonies during reprogramming also displayed mutually exclusive dynamics as determined by *in situ* immunostaining. The proportion of PRDM14(+)OTX2(–) colonies in OG(+) colonies declines from 93% on day 10 to 7% and 0% on day 13 and day 16, respectively; while the proportion of PRDM14(–)OTX2(+) colonies in OG(+) colonies increased from 0% on day 10 to 7% and 90% on day 13 and day 16, respectively; and the proportion peak of PRDM14(+)OTX2(+) colonies were observed on day 13 (Figs. 4H and 4I). Together, these data revealed that the successful branch of SSC reprogramming followed the typical features of reversed developmental trajectory during embryonic development, i.e., spermatogonia to PGCs and further to the epiblasts.

### The intermediate stages and key regulators during SSC reprogramming

Since RPG1/2/3/4 were identified as the pluripotent intermediated stages during SSC reprogramming, DEGs were analyzed for key regulators in these four clusters. Transcription factors (*Rhox5*, *Prdm14*, *Nr0b1*, and *Otx2*) and pluripotency-related genes (*L1td1*, *Utf1*, *Nanog*, and *Dppa3*) were significantly enriched in RPG1/2/3/4 cells (Fig. 5A; Table S6). Furthermore, transcription factor (TF) activity assessed by single-cell regulatory network inference and clustering (SCENIC) (Aibar et al., 2017; Van de Sande et al., 2020), identified regulons related to ‘stem cell proliferation and maintenance’, ‘reproductive structure development’ and ‘epithelial to mesenchymal transition’ in RPG1 to RPG4, which was in line with the features of *in vivo* germ cell development (Figs. 5B and S5A). Of note, *Rhox5*, an essential TF for PGC differentiation (Neri et al., 2013), initiated its expression in RPG1; *Otx2* had been identified as a roadblock to limit entry of epiblast cells to the PGC in a precise temporal window (Zhang et al., 2018). Furthermore, *Rhox5* related genes, and target genes of *Prdm14* as well as *Otx2* were also activated in RPG1, RPG2, and RPG3/4, respectively (Fig. 5A and 5C; Table S6).

To examine the functions of the *Rhox5*, *Prdm14*, and *Otx2* in SSC reprogramming, the CasRx system was used to knockdown these genes in SSCs followed by reprogramming (Fig. 5D, Table S4) (Konermann et al., 2018). Since the expression of *Rhox5*, *Prdm14*, and *Otx2* was extremely low in mouse SSCs, the CasRx based gene knockdown was first validated in gPSCs on transcription and translation levels (Fig. S5B and S5C). Two independent knockdown SSC lines (nearly 50%–80% knockdown efficiency) targeting *Rhox5*, *Prdm14*, or *Otx2* were established respectively without affecting their proliferation and SSC characteristics (Fig. S5D–G). However, after reprogramming the numbers of OG(+) colonies were all dramatically decreased in *Rhox5*, *Prdm14*, or *Otx2* knockdown groups, compared to the non-targeting control (Figs. 5E and S5H), moreover, the PRDM14^+^, TFAP2C^+^, DPPA3^+^ and OTX2^+^ signals were significantly decreased in OG(+) colonies derived from *Rhox5*-knockdown SSCs, compared to control SSCs (Figs. 5F and S5I). These data indicated critical roles for *Rhox5*, *Prdm14*, and *Otx2* in SSC reprogramming.

### Global DNA demethylation and erasure of methylation of imprinting genes in the early stage of SSC reprogramming

It is known that DNA methylation reprogramming is a representative and essential event in germline development (Wang et al., 2014). To explore DNA methylation dynamics, 57 cells treated by DMSO or 5C were selected for single-cell whole-genome bisulfite sequencing (scWGBS) by scTrio-seq2 (single-cell triple omics sequencing 2) to determine the DNA methylation dynamics during SSC reprogramming (Figs. 6A, S4C, and S6A–C; Table S1) (Bian et al., 2018). Integrative analysis showed a correlation between promoter DNA methylation and gene expression in SSC reprogramming, particularly for genes related to the activation of pluripotency as well as PGC-related genes (*Oct4*, *Dppa3*, *Nanog*, *Prdm14*, *Zscan10*, *Gdf3*, and *Fbxo15*) and repression of spermatogenesis-related genes (*Dazl*, *Rhox13*, *Tm9sf5*, *Trim52*, *Sohlh2*, and *Rpl10l*) in RPG2/3/4 (Figs. 6B, S6D and S6E; Table S7).

Hierarchical clustering and multidimensional scaling were performed, and our data were integrated with the DNA methylome profiles of E6.5 epiblast cells, *in vivo* germ cells from E9.5 to E16.5, and sperm (Seisenberger et al., 2012; Wang et al., 2014). From the perspective of the DNA methylome, RPG2 and RPG3 were categorized as comparable to PGCs; while RPG4, SSCs and sperm were clustered into a subgroup by multidimensional scaling (MDS) (Figs. 6C, S6F and S6G). Importantly, the level of global DNA methylation first declined from SSCs to RPG2/3, and was then elevated in RPG4, which was comparable to the *in vivo* germ cell DNA methylation dynamics that occurs between PGC E9.5 and E16.5 stages (Fig. 6D). Genomic features, including promoters, retrotransposons, and intragenic regions, were all demethylated in RPG2/3 and methylated in RPG4 (Figs. 6E, S6C and S6H). This indicated that genomic DNA was globally demethylated and then re-methylated during the RPG1/2/3/4 stages, and a feature of the 5C-RPGs was the enhanced demethylation that more closely matches the *in vivo* PGC development (Figs. 6D, 6E, and S6H). Importantly, we noticed that both paternal and maternal ICRs were also demethylated in RPG2/3, compared to SSCs and RPG1 (Figs. 6F, 6G, and S6I), which mirrored the imprinting erasure seen during *in vivo* PGC development. The global and imprinting demethylation as well as the expression of “chromosome organization”, “pluripotent network”, and “histone modification” related genes of RPG2/3 were more robust in the 5C group than in the control cells, suggesting the importance of thoroughly DNA demethylation and its underlying regulatory mechanisms in the early stage for SSC reprogramming (Figs. 6D–H and S6H, S6I, Table S8).

### Global methylation and *de novo* establishment of imprinting in the late stage of SSC reprogramming

Though RPG2/3/4 were enriched with pluripotent characteristics in the early stage of SSC reprogramming, early-passage gPSCs were more similar to RPG2/3 than to RPG4, according to transcriptome- and DNA methylome features, suggesting that RPG2/3 were more likely to be the initial cells for the late stage of reprogramming (Fig. S7A and S7B). To reveal the DNA methylation dynamics during late stage of SSC reprogramming, SSCs, early- and late-passages gPSCs/5C-gPSCs, and ESCs cell were collected to perform bulk PBAT bisulfite sequencing. Global DNA methylome clustering showed that late-passage 5C-gPSCs were more similar to ESCs compared to gPSCs and early-passage 5C-gPSCs (Figs. 7A, S7C and S7D). Interestingly, we also observed that the global methylation level of 5C-gPSCs significantly increased from early-passage to late-passage during prolonged culture, reaching levels equal to ESCs; however, gPSCs did not acquire this methylation level resembling ESCs even at 25 passages (Figs. 7B, S3F and S7E). Furthermore, *de novo* DNA methyltransferase DNMT3B, DNA methyltransferase DNMT1 and its cofactor UHRF1 also markedly increased their expression in 5C-gPSCs than in gPSCs during prolonged cultivation (Fig. 7C), setting the stage for DNA methylation establishment during the late stage of SSC reprogramming. This indicates that the DNA methylation superiority in 5C-gPSCs over gPSCs persists even in prolonged culture (Figs. 7A–C, S3F, and S7D–E).

Next, DNA methylation dynamics of parental ICRs were carefully investigated. Indeed, in gPSCs the methylation of most maternal ICRs (including *Igf2r*, *Snrpn*, *Impact*, *Peg3*, *Peg10*, and *Plagl1*) declined and remained low in prolonged culture, whilst imprinting methylation at the same ICRs was elevated in 5C-gPSCs, giving the 5C-gPSCs a more ESC-like maternal imprinting pattern than gPSCs (Fig. 7D and 7E). As for paternal ICRs, both gPSCs and 5C-gPSCs maintained nearly 50% DNA methylation levels during the prolonged cultivation (Figs. 7D and S7F). Moreover, when we examined ICRs using individual bisulfite-converted reads, CpG sites in 5C-gPSCs and gPSCs were either near fully methylated or unmethylated in a read-dependent manner (such as *Peg3* and *H19*), indicating the conversion of both maternal and paternal ICRs from the biallelic unmethylation in SSCs to ESC-like monoallelic methylation status in 5C-gPSCs and gPSCs (Fig. S7G). Impressively, allele split further verified the re-methylation of maternal ICRs (*Impact*, *Peg10*, and *Snrpn*) of one of the two alleles in one cell during the late stage of reprogramming, showing the *de novo* establishment of imprinting status in SSC reprogramming (Fig. 7F and 7G).

The poor developmental potential of CiPSCs inspired us to examine their DNA methylome and imprinting status (Fig. S7H; Table 1). Compared to ESCs, early passage CiPSCs (p5) were globally hypomethylated on distinct genomic features, including both paternal and maternal ICRs, suggesting extreme methylation erasure or incomplete epigenetic reprogramming in CiPSCs (p5) (Figs. 7H, 7I and S7E). These demethylation features of paternal ICRs (including *H19*, *Rasgrf1*, and IG) and maternal ICRs (including *Igf2r*, *Gnas1a*, *Peg1/Mest*, *Gnas*, *Peg3*, *Snrpn*, *Peg10*, and *Peg13*) were observed in three CiPSC lines (Fig. S7I). Moreover, DD regions and the included miRNAs encoded locus in two CiPSC lines (R-1 and K-2, p5) remained de-methylated even with long-term expansion in 2iL (p25), which provided hints for the correlation between aberrant DNA methylation loss of the whole genome or imprinting locus and the defective pluripotency of CiPSCs (Fig. S7J and S7K), which was similar to that of gPSCs, emphasizing the pivotal role of precise epigenetic reprogramming during cell fate transitions, and their biological functions.

Together, SSCs were first globally DNA demethylated and then re-methylated to an ESC-like pattern during spontaneous reprogramming with 5C induction, especially at imprinted genes acquiring mono-allelic methylation. That is, in an individual 5C-gPSC, for the ICRs of imprinting genes, one allele was fully methylated whereas the other allele was completely unmethylated. This effect was attained in a reversed germline differentiation trajectory and correlated with the true pluripotency of 5C-gPSCs (Fig. 7J).

## Discussion

In mammals, germ cells carry the genetic inheritance of each individual and the epigenetic information required to produce the next generation. The cell fate reprogramming of germ cells is a fascinating question in reproductive and developmental biology. Previous studies have reported that SSCs have the potential to spontaneously reprogram to pluripotent stem cells (Kanatsu-Shinohara et al., 2004; Guan et al., 2006); however, the underlying mechanisms are largely unknown. In this study, using single-cell transcriptome and DNA methylome sequencing we provide a high-resolution landscape that uncovers the unique reprogramming trajectory and regulatory networks in SSC reprogramming.

Building on this data, we developed a five-chemical combination that targeted metabolic and epigenetic regulatory pathways to improve reprogramming efficiency about 80–100 times, which was more effective than previous reported genetic manipulation by gene overexpression (*Oct4*), gene knockdown (*Dnmt1*, *Dmrt1*, *Trp53*, or *Zeb1*) or signaling pathway inhibition (TGF-b) (Takashima et al., 2013; An et al., 2017). This supported our purpose to control the epigenetic remodeling and metabolic regulation that is critical for SSC reprogramming, which has not been explored in any previous attempts to improve SSC reprogramming efficiency. Moreover, we defined a large proportion of SSCs with fibroblast, meiosis, early-pluripotency, and neural features as the failed branches which helps explain the low efficiency of SSC reprogramming.

gPSCs and gPSCs derived from chemical induction in our study can both produce mice by tetraploid complementation but had not achieved in gPSCs established by Kanatsu-Shinohara et al. We speculated that some probabilities led to this capability: (1) The gPSCs developed by Kanatsu-Shinohara et al. were tetraploid complementation competent, but the number of embryos reconstructed by gPSCs may not be enough, as viable 4N-mice from ESCs was not reported in that study (Kanatsu-Shinohara et al., 2004); (2) SSC reprogramming in our study occurred after long term cultivation without splitting, rather than regular SSC cultivation protocol as Kanatsu-Shinohara et al. used, suggesting the importance of unique reprogramming trajectory and regulatory network underlying the present reprogramming environment. Most importantly, the low efficiency to obtain 4N-comp mouse from gPSCs and severe mortality of gPSC 4N-comp mouse suggested the incomplete reprogramming of gPSCs from SSCs. Whereas, the significant differences between 5C-gPSCs and gPSCs to producing 4N-comp mice also highlights the fully reprogramming of 5C-gPSCs as well as the critical role of 5C induction in SSC reprogramming.

Interestingly, compared with the primitive streak-like intermediates and two-cell like (2C-like) cells during OSKM-iPSCs and CiPSCs, respectively (Takahashi et al., 2014; Zhao et al., 2018), we identified spermatogonia-, PGC-, and epiblast-like cells as key intermediate stages during SSC reprogramming, this contrasts with the appearance of other intermediate cells in somatic cell reprogramming. Here, we show that gPSCs progress backwards through the normal developmental pathway, rather than adopting a novel cell type conversion program, for example, only 2C-like cells but not a developmental route appear in chemical reprogramming. Indeed, *Rhox5* has been shown to be modulated by DNMT3L in PGC development (Neri et al., 2013), coincidentally, we show that *Rhox5* is also functionally required in SSC reprogramming. This strongly supports a ‘reversed trajectory’ for the reprogramming of SSCs to gPSCs, exactly mirrored the normal *in vivo* developmental trajectory, as they pass through the same intermediate stages and even have the same molecular requirements, but in the reversed order. This contrasts with normal somatic reprogramming, where many reprogramming factors are essential for reprogramming, but are dispensable in differentiation (Zhuang et al., 2018).

DNA methylation signatures at imprinted genes are only removed with the switch from monoallelic methylation in the inner cell mass (ICM) and E6.5 epiblasts to hypomethylation in E13.5 PGCs. This allows the removal of the parental epigenetic ‘memory’ and subsequent imprinting re-establishment in spermatogonia and immature oocytes, which establish biallelic methylation of both alleles at most ICRs (Seisenberger et al., 2012; Gu et al., 2019). Along with the erasure and acquisition of global methylation during the early and late stage of SSC reprogramming, respectively, imprinted locus in SSCs were symmetrically methylated on both alleles and converted to an asymmetric re-methylated status on one of the two alleles in 5C-gPSCs. This newly identified imprinting re-methylation of one of the two alleles in the late stage of SSC reprogramming has not been previously observed in any *in vivo* developmental processes or *in vitro* reprogramming, as DNA re-methylation on both alleles in spermatogonia and immature oocytes, and imprinting maintenance in somatic cell differentiation as well as reprogramming (Reik et al., 2001). Moreover, it was interesting as 5C treatment was only applied to SSCs undergoing reprogramming in the early stage (Fig. 2C; days 0 to 19), yet implying the indirectly impact of the 5C combination even in the late stage of reprogramming (Fig. 7D and 7E). This provides a new angle to understand as well as a novel strategy to manipulate reprogramming of imprinting genes (Fig. 7J).

Previous studies have shown that genomic imprinting plays a pivotal role in the growth, viability, and various physiological functions of mammalian embryos (Tucci et al., 2019). We showed that imprinting defects were much less in 5C-gPSCs than gPSCs, and provided an explanation for the reduced developmental and growth disorders observed in embryos and pups derived from 5C-gPSCs than gPSCs. Accordingly, CiPSCs had limited developmental potential and global imprinting loss. Due to the importance of correct imprinting in PSCs, it was important to further dissect the underlying regulatory mechanisms behind *de novo* establishment of imprinting status during SSC reprogramming. Potentially, this may bring insights into the mechanisms beyond accurate imprinting erasure and re-establishment, which remains somewhat unclear. Additionally, fine-tuned control of imprinting may help overcoming epigenetic disorders to improve other cell fate decisions, and rescue imprinting aberrations from uniparental disomy. Indeed, in addition to the epigenetic disorders of CiPSCs, recent studies have revealed that the mutation rate in somatic cells is much higher than that originated from germline cells (Moore et al., 2021; Yang et al., 2021), suggesting that SSC reprogramming would be a safer strategy to obtain pluripotent stem cells with less mutations than somatic cells reprogramming.

Overall, we systematically dissect SSC reprogramming at single cell resolution, and based on the rational application of chemicals we can drastically improve the efficiency up to 80- to 100-folds, and in the process our approach also repairs the poor developmental potential of gPSCs. The conversion of SSCs to pluripotent stem cells is accompanied by epigenetic reprogramming which mirrored *in vivo* germ cell development. Finally, this unique trajectory and regulatory network of SSC reprogramming described here expands our understanding of cell fate reprogramming, such knowledge paves the way to obtain high-quality iPSCs without genetic manipulation for future clinical applications in regenerative medicine.

## Materials and methods

### Animal model and ethics statement

C57BL/6 and KM mice were from the laboratory animal center of Southern Medical University, *Oct4*-EGFP and CAG-EGFP transgenic C57BL/6 mice and DBA/2 mice were purchased from Nanjing Biomedical Research Institute of Nanjing University. *Oct4*-EGFP transgenic mice express Enhanced Green Fluorescent Protein (EGFP) under the control of the POU protein domain, class 5, transcription factor 1, promoter and distal enhancer. ICR mice were purchased from Guangdong Medical Laboratory Animal Center.

### Establishment of gPSCs, CiPSCs, and cell culture of PSCs

B6D2F1 SSCs were derived from the 5.5 days postpartum (dpp) male mice generated by mating *Oct4*-EGFP or EGFP transgenic C57BL/6 female with DBA/2 male. SSCs derivation and expansion were performed according to the previously study (Wang et al., 2021), with culture medium containing 20 ng/mL mouse epidermal growth factor (EGF) (R&D), 10 ng/mL human basic fibroblast growth factor (bFGF) (R&D), 10 ng/mL rat glial cell line-derived neurotrophic factor (R&D) and 10^3^ U/mL ESGRO (Sigma-Aldrich). gPSCs were established by following Ko et al.’s protocol (Ko et al., 2009), briefly, approximately 4,000 single cells of SSCs were plated on one well of 6 µg/mL fibronectin (Millipore) coated 24-well plate containing mitomycin C-inactivated mouse embryonic fibroblast (MEF) feeder cells in SSC culture medium, the medium was changed every 3 days. OG(+) colonies could be observed within 2 to 4 weeks.

R-1 CiPSCs were established from MEF of *Oct4*-EGFP transgenic mice (C57BL/6 background) as previous study (Zhao et al., 2015). K-2 CiPSCs (C57BL/6 background) were gifted from Liu lab (Cao et al., 2018). D-1 CiPSCs were reprogrammed from MEF of *Oct4*-EGFP transgenic mice (B6D2F1 background) with an efficient protocol (Zhao et al., 2018). ESCs were established from *Oct4*-EGFP blastocysts (B6D2F1 background) according to previous protocol (Ying et al., 2008). gPSCs, 5C-gPSCs, ESCs, and CiPSCs were maintained on feeder layers in ground state ESC culture medium N_2_B_27_ supplemented with 2iL (3 µmol/L CHIR99021, 1 µmol/L PD0325901, and 10^3^ U/mL ESGRO). SSCs and PSCs were maintained at 37°C in an atmosphere of 5% CO_2_ in air.

### Isolation and collection of SSCs, reprogramming cells and gPSCs for single-cell profiling

Four thousand two hundred forty-seven and 1,089 single cells during SSC reprogramming and SSCs as well as PSCs were collected for scRNA-seq and scTrio-seq2, separately. SSC- and PSC-like colonies were selected and digested in 0.05% trypsin for 3 min, followed by inactivation of trypsin by DMEM (ThermoFisher) (containing 10% FBS). After centrifugation, these cells were suspended in DMEM (containing 10% FBS) for further sample selection by using a mouth pipette to transfer single cells into prepared lysis buffer with an 8-nt barcode.

### scRNA-seq library preparation and sequencing

The preparation of single cell RNA-seq library was performed using the modified single-cell tagged reverse transcription sequencing (STRT-seq) protocol (Dong et al., 2018). Briefly, the single cell was lysed and add 2.85 μL reverse transcription (RT) mix containing 500 U SuperScript II reverse transcriptase (Invitrogen), 4 U RNase inhibitor (TAKARA), 30 mmol/L MgCl_2_, and 5 µmol/L TSO primer, followed by incubation at 25°C for 5 min, 42°C for 60 min, 50°C for 30 min and 70°C for 10 min. Then the cDNA amplification and purification by 0.8× AMPure XP beads (Beckman) was performed. Biotin PCR was further carried out and enriched. Finally, the single cell RNA-seq library was prepared according to the Hyper Prep Kits with PCR Library Amplication/Illumina series (KAPA). The libraries were checked and sequenced with 150 bp pair-end reads on Illumina Hiseq 4000 (Novogene).

### Processing of scRNA-seq data

We firstly extracted cell barcodes and UMI sequences from read 2 of raw fastq data by UMItools (v1.0.0) (Smith et al., 2017) and attached them after the read name of read 1. After TSO and polyA sequence removal by local scripts, the ‘clean’ reads were aligned to the mouse mm10 reference genome using STAR (2.7.2b) (Dobin et al., 2013), gene features were assigned by featureCounts (v2.0.0) (Liao et al., 2014). We used UMItools to generate UMI count expression matrix.

For the quality control of scRNA-seq data, cells with detected gene numbers fewer than 2,000 or the fraction of mitochondrial reads more than 5% were excluded from further analysis, additionally, genes expressed in less than 3 cells were filtered out of the analysis.

### Dimensional reduction and cell clustering

Seurat (v3.1.3) (Butler et al., 2018; Stuart et al., 2019) was used for principal component analysis (PCA) and UMAP dimensional reduction of scRNA-seq data. Cell clusters were detected by shared nearest neighbor (SNN) modularity optimization-based clustering algorithm using Seurat function ‘FindClusters’ with default parameters. Cell types were assigned based on the expression of canonical marker genes and the differential expressed genes (DEGs) of each cluster. For scRNA-seq of 5C and DMSO-induced SSC reprogramming, cell types identification by scPred with a machine-learning probability-based prediction method (Alquicira-Hernandez et al., 2019). In detailed, scRNA-seq data of SSC reprogramming from control system were used to build reference object and train cell-type classifier with default parameters. The query data of 5C and DMSO scRNA-seq were then aligned to reference and classified with the ‘scPredict’ function, respectively. The prediction processes were repeated 500 times with different random seeds for each dataset, and the cell-type predicted the most times for each cell was assigned as cell type labels.

### Single-cell trajectory analysis

We used three different algorithms to infer the trajectory of SSC reprogramming, including PAGA (partition-based graph abstraction) (Wolf et al., 2019), RNA velocity (La Manno et al., 2018), and Monocle2 (Trapnell et al., 2014). PAGA was performed in SCANPY (1.4.4) (Wolf et al., 2018) by converting Seurat object into a loom format. SNN graph and UMAP embedding calculated in Seurat were used to map the coarse-grained structures. For RNA velocity, command line interface of velocyto.py was used to generate spliced and un-spliced expression matrices from aligned bam files, and then the RNA velocity of each cell were estimated by velocity.R and visualized in UMAP plot. Monocle2 trajectories were constructed according the default workflow, top 2,000 highly variable genes identified from Seurat procedure were used to construct the trajectory of all clusters in the early stage of SSC reprogramming related to Fig. S2B. Top 100 DEGs of each cluster were used to construct trajectory of successful reprogramming branch (including SSC1-4 and RPG 1-4) related to Fig. 1G.

### Identification of differentially expressed genes and GO analysis

Differential expressed genes (DEGs) of all clusters were found by Seurat ‘FindAllMarkers’ function with parameter ‘logfc.threshold = 0.5’. Only DEGs with adjusted *P*-value < 0.05 were remained. DEGs among clusters were found by Seurat ‘FindMarkers’ functions with the same criterion. Gene ontology (GO) enrichment analysis was performed using Metascape (Zhou et al., 2019).

### Gene signatures

For better identify and describe the characteristic of cells, we constructed gene sets and scored cells based on their expression of these genes, as previous described by Schiebinger et al. (2019). In detail, the signature used in Fig. S2D was constructed from top 100 upregulated DEGs of SSCs (SSC signature), ESCs (pluripotency signature) and other corresponding clusters. In Fig. S2H, ‘Glycolysis/Gluconeogenesis’ signature was derived from KEGG pathway mmu00010, ‘DNA transmethylase & demethylase’ signature included *Dnmt1*, *Dnmt3a*, *Dnmt3b*, *Dnmt3l*, *Tet1*, *Tet2*, *Tet3*, and *Tdg*. The pluripotency signature was defined by the expression of a set of canonical pluripotency genes (Table S2).

### Transcriptional comparison between SSC reprogramming and fibroblast reprogramming (CiPSC and OSKM-iPSC)

RPG1 of SSC reprogramming (this study), cells at day 10 of stage III during CiPSC induction (Zhao et al., 2018), and cells at day 6 of OSKM-iPSC reprogramming (Schiebinger et al., 2019) initiating their expression of pluripotent-related genes were designated as key intermediate states in each reprogramming process. Stages 1 and 2 of each reprogramming process were divided by the above-mentioned intermediate states. DEGs and GO term analysis were performed and compared among these three distinct reprogramming processes in stages 1 and 2, respectively.

### Immunofluorescence and alkaline phosphatase staining

For immunofluorescence staining, the cells were cultured on coverslips and fixed with 4% paraformaldehyde for 30 min and then permeated with 0.3% Triton X-100 for 15 min followed by blocking with 5% BSA (Sigma-Aldrich) at room temperature. Afterwards, cells were incubated with primary antibodies at 4°C overnight and secondary antibody at room temperature for 1 h. The nuclei were counterstained with 10 μg/mL Hoechst 33342 (Thermofisher) for 15 min at room temperature. Images were captured by confocal microscope (ZEISS LSM880). Alkaline phosphatase staining was performed with BCIP/NBT Alkaline Phosphatase Color Development Kit (Beyotime) according to manufacturer’s instructions. Images were captured with stereomicroscope (Zeiss, Axio Zoom. V16).

The primary antibodies used in this study were listed: Goat anti-GFRa1 (R&D, AF714), Mouse anti-ZBTB16 (Santa, sc-28319), Rabbit anti-DDX4 (Abcam, ab13840), Mouse anti-POU5F1 (OCT4) (Abcam, ab19857), Mouse anti-POU5F1 (OCT4) (Santa, sc-5279), Mouse anti-SSEA1(Sigma-Aldrich, MAB4301), Rabbit anti-NANOG (Abcam, ab70482), Rabbit anti-RHOX5 (Abcam, ab31922), Rabbit anti-PRDM14 (Cell Signaling Technology, 83527), Goat anti-OTX2 (R&D, AF1979), Rabbit anti-DPPA3 (Abcam, ab19878), Rabbit anti-L1TD1(Biorbyt, orb35537), Mouse anti-TFAP2C (Santa, sc-12762), Rabbit anti-DNMT3B (proteintech, 26971-1-AP), Rabbit anti-DNMT1 (abclonal, A16729), Mouse anti-UHRF1 (Santa, sc-373750), and Mouse anti-ACTIN (proteintech, CL594-66009). The secondary antibodies (Jackson ImmunoResearch) used in this study: Goat Alexa Fluor 594 anti-rabbit IgG (111-585-003), Goat Alexa Fluor 488 anti-rabbit IgG (111-545-003), Goat Alexa Fluor 647 anti-mouse IgG (115-605-003), Goat Alexa Fluor 594 anti-mouse IgG (115-585-003), Goat Alexa Fluor 594 anti-goat IgG (705-585-003), and Donkey Cy2 anti-goat IgG (705-225-147). Enhanced chemiluminescence peroxidase-labeled anti-mouse or rabbit antibodies (ZSGB-BIO, ZB-2305 or ZB-2301) were used for western blotting analysis. The intense analysis was performed by ImageJ (National Institutes of Health).

### Teratoma formation

Teratoma formation was performed as previously described (Zhao et al., 2009). In brief, 1 × 10^6^ cells of gPSCs (gPSC-1 p10) and 5C-gPSCs (5C-gPSC-1 p10) were suspended at 100 µL PBS, respectively. These cells were subcutaneously injected into the inguen of 8-week-old male NSG mice. Six to 8 weeks later, injected mice were sacrificed and teratomas were fixed with 4% paraformaldehyde. Paraffin sections were stained with hematoxylin and eosin.

### Extracellular lactic acid analysis and 2-DG treatment

Extracellular lactate content was measured by the Lactate Assay Kit (Sigma-Aldrich). Standard curves were plotted as manufacturer’s instruction. Supernatants without insoluble material during SSC reprogramming were collected for subsequent reaction with Lactate Enzyme mix and Lactate Substrate Mix at room temperature avoid from light for 30 min. Absorbances at 450 nm (*A*_450_) of each sample were measured by an automated micro plate reader (TECAN®infinite M200). SSCs were treated with 2-DG (Target Mol) during reprogramming, and the concentration of 2-DG was applied as previously described (Kanatsu-Shinohara et al., 2016).

### Chemical screening

Epigenetics Compound library (Target Mol) and some in-house chemical collections were applied for the chemical screening (Table S4), each individual compound at 10 µmol/L concentration was added into each well with three repeats for the first-round screening. From 10 days after treatment, the number of OG(+) colonies was counted under a fluorescence microscope to evaluate the reprogramming efficiency of each chemical. After the first-round screening, concentration test and compound combination were further performed for a high-efficient combination.

### Diploid blastocyst injection and tetraploid embryo complementation

The generation of mice by diploid blastocyst injection and tetraploid embryo complementation was carried out as previously described (Zhao et al., 2009). For diploid blastocyst injection, 10 to 15 pluripotent stem cells (B6D2F1 genetic background, black coat color) were injected into each diploid blastocyst collected from ICR female mice. For tetraploid embryo complementation, two-cell stage embryos were collected from oviducts of ICR females (white coat color) and electrofused by Electro Cell Manipulator (BTX, EMC2001) to produce one-cell tetraploid embryos that were then cultured in M2 medium (Sigma-Aldrich). Eight pluripotent stem cells were injected into each tetraploid blastocyst. Every 16 injected blastocysts were transplanted into one ICR pseudopregnant recipient female. Embryos derived from diploid- and tetraploid-blastocyst injection were dissected on E12.5 and the day of birth (E19.5), respectively. Fluorescence and bright field images of chimeric embryos were captured by stereomicroscope (Zeiss, Axio Zoom.V16).

### Determination of the SSLP by PCR

The SSLP determination was performed as previously described (Zhou et al., 2001). Sequences for the primer pairs were found on the Mouse Genome Informatics website. DNA was extracted from mouse tail tips with DNA Isolation Mini Kit (Vazyme). PCR Products were separated by 3% agarose gels and visualized by ethidium bromide staining. Primers were listed in Table S4.

### Integrative and comparative analysis of scRNA-seq data of SSC reprogramming and *in vivo* germ cell development

In order to compare the transcriptional signature of SSC reprogramming process and *in vivo* mouse germ cell development, the Harmony (v1.0) algorithm (Korsunsky et al., 2019) was used to integrate these two datasets through Seurat function ‘RunHarmony’ with default parameters. scmap (v1.1.6) (Kiselev et al., 2018) was used to perform cell types projection between datasets of the successful branch of SSC reprogramming and *in vivo* germ cell development.

### Derivation of transgenic SSC lines

Gene knockdown was performed by using CRISPR/CasRx system for targeting *Rhox5*, *Prdm14*, and *Otx2*. Lentivirus packaging and transfection were performed as previously described (Konermann et al., 2018; Wessels et al., 2020). Stably transfected SSCs were purified by the addition of puromycin (Thermofisher) and zeocin (Thermofisher) at a final concentration of 0.4 and 15 μg/mL for about 1 week, respectively. gRNA sequences were listed in Table S4.

### Quantitative PCR (q-PCR) analysis

PSCs were lysed in Trizol reagent (TIANGEN), and total RNA was isolated using Chloroform extraction and treated with DNase. cDNA was synthesized using HiScript® II Reverse Transcriptase (Vazyme). q-PCR was performed using 2× RealStar Green Fast Mixture (GenStar) on LightCycle® Real-Time PCR System (Roche). The data were analyzed using the delta-delta Ct method. Geometric mean of *Rps2* and *Gapdh* was used as an inner control to normalize the expression of target genes. Primers were listed in Table S4.

### Transcription factor analysis

We performed pySCENIC (single-cell regulatory network inference and clustering) (Aibar et al., 2017; Van de Sande et al., 2020) analysis to identify activated TFs and regulatory sub-network of each TF (regulons) for successful branch of SSC reprogramming. For TFs not included in SCENIC’s TFs list, we identified TFs according to TFs list from TcoF-DB (Schmeier et al., 2017) and constructed regulons based on PPI information of STRING database as supplementation. TF-targets network constructed from SCENIC were visualized by Cytoscape (v3.9.1) (Shannon et al., 2003).

### Single-cell Trio-seq2 sequencing

scTrio-seq library was constructed following previously reported modified scTrio-seq2 protocol (Bian et al., 2018). In details, each single cell was picked into lysis buffer containing magnetic beads (Invitrogen). Single cells were lysed and vortexed for 1 min to release RNA, and placed on the magnetic rack for 5 min. Supernatants containing RNA were transferred to a new tube for scRNA-seq, nuclei aggregated with magnetic beads were maintained in the pellet and re-suspended with lysis buffer of scBS-seq for DNA methylation sequencing. Bisulfite conversion was conducted by EZ-96 DNA Methylation-Direct MagPrep kit (Zymo). Four rounds of amplification were performed with Klenow exo- (ENzymics) and scBS-seq-P5-N6-oligo1 (CTACACGACGCTCTTCCGATCTNN-NNNN). After purification by 0.8× AMPure XP beads, the products were used to synthesize the second strand using scBS-seq-P7-N6-oligo2 (AGACGTGTGCTCTTCCGATCTNNNNN). 16 cycles of PCR program were subsequently performed to complete the DNA library construction incorporated with universal primers and index primers (New England Biolabs), followed by twice purification with 0.8× AMPure XP beads. DNA libraries were sequenced on the Illumina Nova platform with 150 bp paired-end reads.

### Post-bisulfite adaptor tagging (PBAT) library preparation

PBAT was performed as the published protocols (Smallwood et al., 2014). Briefly, genomic DNA was extracted using FastPure Cell/Tissue DNA Isolation Mini Kit (Vazyme, DC102). Then 5 ng isolated genomic DNA together with 60 pg unmethylated lambda DNA (ThermoFisher) were subjected to bisulfite conversion, followed by column-based purification (Zymo, D4014). Next, random priming, library amplification and subsequent sequencing were conducted on the bisulfite-converted DNA for single-cell DNA methylation library preparation described above.

### Bisulfite sequence data process

Trim_galore (v0.6.4) was used to remove low quality reads and adaptor sequence, and 9 bp random primers from 5ʹ end of both read 1 and read 2 were also trimmed. Clean reads were then mapped to mm10 genome using Bismark (v0.23.0) with paired-end mode and ‘--non_directional’ option. Unmapped reads were outputted and mapped again by single-end mode. Aligned bam files were merged and sorted by samtools (v 1.11) and PCR duplicates were marked and removed by picard MarkDuplicates (v2.23.6). Methylation calls for each CpG site were extracted by ‘bismark_methylation_extractor’ of Bismark. The thresholds for CpG sites coverage were 1× and 3× for single-cell and bulk DNA methylome, respectively. Only methylation levels less than 10% or greater than 90% were retained for single-cell methylation data. Bisulfite conversion rate (BSCR) was estimated by methylation ratio of lambda DNA, samples with BSCR greater than 98% and detected CpG sites greater than 1,000,000 were kept for further analysis. BedGraph files containing methylated and unmethylated sites were generated by local scripts and converted to bigwig format by UCSC binary tools ‘bedGraphToBigWig’ (v4).

### Methylation levels of genomic elements and differentially methylated promoters

The annotation of known genomic elements was downloaded from UCSC genome browser, including RefSeq genes, CGI, enhancers and so on. The annotations of transposable elements were obtained from RepeatMasker of UCSC table browser. Promoters were defined as the 2-kb upstream and downstream of transcription start sites (TSS) according to RefSeq gene annotation. The methylation level of each genomic element was calculated by the average methylation level of all covered CpG sites (at least 3 CpG sites). The global methylation level of each sample was measured by the average methylation level of all the genomic 1-kb windows (at least 3 CpG sites). The scaled methylation levels of RefSeq genebodies and CGIs (CpG islands) were calculated by the ‘computeMatrix’ command of deeptools (v3.4.3) in scale-region mode and visualized by local scripts. Differentially methylated promoters (DMPs) with methylation difference greater than 30% and *P* value <0.05 were found in every two clusters by multiple *t*-test.

### Clustering of global methylation

To compare the global methylation level of distinct samples, the average methylation levels of all 1-kb tiles covered by more than 80% samples were applied to calculate Euclidean distance among samples by ‘dist’ function of R ‘stats’ package. Distance matrix was used to perform hierarchical clustering by ‘hclust’ function. We also performed multidimensional scaling (MDS) analysis with the Euclidean distance and projected it in two-dimensional space.

### Correlation analysis between gene expression and promoter DNA methylation

To calculate the correlation between gene expression and DNA methylation during SSC reprogramming, average gene expression levels and promoter DNA methylation levels derived from Trio-seq2 data were calculated for each gene in each cluster of successful branch, respectively. Pearson correlation coefficient was then determined by ‘cor’ function with default parameter in R.

### Methylation of allele-specific imprinting control regions

Known imprinting control regions (ICRs) were annotated and the methylation level of each ICR were calculated as previously mentioned. To investigate the allele-specific methylation of ICRs, SNPsplit (v0.4.0) (Krueger and Andrews, 2016) was used to determine the allelic origin of distinct reads via known SNP positions. In detail, SNP information for C57BL/6NJ and DBA/2J (dbSNP142) were downloaded from MGP websites. Dual strain reference genome was generated according to SNPsplit workflow and the strain-specific SNP positions were masked by Ns. Bisulfite alignment was performed by Bismark again and allele-specific alignments were split, the methylation information of each allele was extracted separately. The methylation calls by reads which covering ICRs were extracted from Bismark’s CpG context-dependent methylation files and lollipop methylation diagrams showing methylation information of each CpG site for distinct reads were generated by local script and visualized with ‘ggplot2’ package in R.

## Supplementary information

The online version contains supplementary material available at https://doi.org/10.1093/procel/pwac044.

pwac044_suppl_Supplementary_Materials

pwac044_suppl_Supplementary_Table_S1

pwac044_suppl_Supplementary_Table_S2

pwac044_suppl_Supplementary_Table_S3

pwac044_suppl_Supplementary_Table_S4

pwac044_suppl_Supplementary_Table_S5

pwac044_suppl_Supplementary_Table_S6

pwac044_suppl_Supplementary_Table_S7

pwac044_suppl_Supplementary_Table_S8

## Data Availability

The scRNA-seq and WGBS data are deposited in the Gene Expression Omnibus (GEO) under the accession code GSE186260. All other data supporting the findings of this study are available from the Lead Contact upon reasonable request. All codes in this study are available upon reasonable request.

## Abbreviations

2-DG: 2-deoxyglucose
2iL: 2 inhibitors and Lif (ground state embryonic stem cells culture medium)
4N-comp: tetraploid complementation assay
5C: 5 chemicals including SGC707, vitamin C, (-)-epigallocatechin gallate, daphnetin, and tauroursodeoxycholic acid
5C-gPSCs: 5 chemicals induced germline-derived pluripotent stem cells
AP: alkaline ahosphatase
Bleo: bleomycin
BSCR: bisulfite conversion rate
CiPSCs: chemically induced pluripotent stem cells
DD region: *Dlk1-Dio3* region
DEGs: differentially expressed genes
DMPGs: differential methylated promoter genes
EGCG: (-)-epigallocatechin gallate
EGFP: enhanced green fluorescent protein
ESCs: embryonic stem cells
FGC: fetal germ cell
GC: germ cell
GO: gene ontology
gPSCs: germline-derived pluripotent stem cells
ICM: inner cell mass
ICRs: imprinting control regions
iPSCs: induced pluripotent stem cells
MDS: multidimensional scaling
OG(+): Oct4- enhanced green fluorescent protein positive
PAGA: partition-based graph abstraction
PBAT: post-bisulfite adaptor tagging
PGC: primordial germ cell
PND: postnatal day
PSCs: pluripotent stem cells
Puro: puromycin
RPG: reprogramming
SCENIC: single-cell regulatory network inference and clustering
scRNA-seq: single-cell RNA sequencing
scTrio-seq2: single-cell triple omics sequencing 2
scWGBS: single-cell whole-genome bisulfite sequencing
SPG: spermatogonia
SSCs: spermatogonial stem cells
SSCM: spermatogonial stem cell culture medium
SSLP: simple sequence length polymorphism
STRT-seq: single-cell tagged reverse transcription sequencing
TES: transcription end site
TF: transcription factor
TSS: transcription start site
TUDCA: tauroursodeoxycholic acid
UMAP: uniform manifold approximation and projection
UMIs: unique molecular identifiers

## Resource availability

Further information and requests for resources should be directed to and will be fulfilled by the lead contact, Xiao-Yang Zhao (zhaoxiaoyang@smu.edu.cn)

## Materials availability

This study did not generate new unique reagents.
