# Supplementary material for: The chemical reprogramming of unipotent adult germ cells towards authentic pluripotency and de novo establishment of imprinting
Source: Protein Cell. 2022 Nov 17;14(7):479–98. doi: 10.1093/procel/pwac044 (PMC10305742; doi:10.1093/procel/pwac044)
Supplement: pwac044_suppl_Supplementary_Materials [file pwac044_suppl_supplementary_materials.pdf]

Figure S1. Pluripotent stem cells derived from mouse spermatogonial stem cells and sample information of RNA-seq Data, related to Figure 1

(A) Immunofluorescence of ZBTB16 (red, top) co-stained with OCT4 (green, top), and GFRA1 (red, bottom) co-stained with DDX4 (pink, bottom) in mSSCs (SSC0606 p10). Scale bar, 20 μm.

(B) Immunofluorescence of DDX4 (red) and PNA (green) in the testicular paraffin sections from *Kit*$^{w/wv}$ mice transplanted with (top) or without (bottom) mSSCs (SSC0606 p10), respectively. Scale bar, 40 μm.

(C) Immunofluorescence of OCT4, NANOG, SSEA1 and DDX4 (red) in gPSCs (gPSC-1 p7). Scale bar, 20 μm.

(D) Metaphase spread of karyotype showing 40 chromosomes of gPSC-1 (p10). Scale bar, 20 μm.

(E) H&E staining of paraffin sections of teratoma, which were formed after subcutaneous injection of gPSC-1 (p15) into nude mice. Endodermal gland structure of gastrointestinal (left); mesodermal muscle fibre (middle); ectodermal cornified layer and keratinocyte (right). Scale bar, 100 μm.

(F) Bright field and fluorescence images showing E12.5 embryos of KM mouse and chimaera embryo produced by blastocyst injection of *CAG*-EGFP gPSC-2 (p10), 4 out of 7 embryos were chimaeras after gPSCs injection. Scale bar, 2 mm.

(G) Bright filed and fluorescence images of the E12.5 genital ridges derived from blastocyst injection of *Oct4*-EGFP gPSC-1 (p10), 5 out of 13 pairs of genital ridges were GFP positive after gPSCs injection. Scale bar, 500 μm.

(H) Chimaeras produced from B6D2F1 (black coat color) gPSC-1.

(I) Summary of chimeric assays of diploid blastocyst injection using gPSC-1.

(J) Sample information of reprogramming cells (original condition), gPSCs, and ESCs for scRNA-seq analyzed in this study.

(K) UMAP plot showing reprogramming cells from two batches of SSC reprogramming, gPSCs and ESCs.

(L) Boxplot showing the numbers of genes (top) and UMIs (bottom) detected in each single cell.

(M) UMAP plot showing reprogramming cells from day 0 to day 27.

(N) Violin plots showing the expression levels (log (TPM/10+1)) of specific genes in each cluster.

Figure S2. Transcriptomic signatures of SSC reprogramming, related to Figure 1

(A) UMAP plot showing the RNA velocity analysis results of SSC reprogramming, each arrow represents the predicted developmental direction of the individual cell.

(B) Cell trajectory of the early stage during SSC reprogramming inferred by Monocle2. Arrows indicate the developmental orders of these cells.

(C) PAGA results visualized by the temporal information of each cluster during the early stage of reprogramming.

(D) PAGA results visualized by the scores of distinct signatures. The color key from dark blue to bright yellow indicates low to high signature scores, respectively.

(E) UMAP plot showing the transcriptome data of the successful and failed branches of SSC reprogramming.

(F) Enriched GO terms of four representative clusters of failed branches.

(G) Violin plots showing the expression dynamics of KEGG pathway 'Glycolysis/Gluconeogenesis' (mmu00010) in the failed branches of SSC reprogramming.

(H) Pearson correlation between signature score of pluripotency-related genes and glycolysis- as well as DNA transmethylase & demethylase-related genes based on gene expression in each single cell amongst RPG1/2/3/4. Genes sets used in each signature could be found in Table S2.

(I) Venn diagram showing DEGs in the stage 1 and stage 2 of three reprogramming processes, corresponding to Figure 1M.

(J) Bubble plots showing GO terms down-regulated in stage 1 (top) and stage 2 (bottom) of three reprogramming processes, corresponding to Figure 1M.

Figure S3. The Developmental Potential of PSCs Estimated by Tetraploid Complementation, the Methylation Level of SSCs and PSCs, related to Figure 3

(A) E12.5 embryos (left) produced by tetraploid complementation (4N-comp) of ESCs, 5C-gPSC-S2, and gPSC-S2, respectively, and their corresponding E12.5 genital ridges (middle and right). Scale bar, 2 mm (left), 500 μm (middle and right).

(B) ESC (top) and gPSC-S2 (bottom) 4N-comp pups and their corresponding placentae. Note, * Pups survived beyond 4 days postnatal; # Pups died within 4 days postnatal.

(C) Bright field, fluorescence images of E3.5 *Oct4*-EGFP blastocysts flushed from an ICR female mouse mated with F1-5 ESC (top), 5C-gPSC-S2 (middle), and gPSC-S2 (bottom) 4N-comp mice, respectively. Scale bar, 100 μm.

(D) An 11-week-old F1-5 ESC 4N-comp mouse with a uniformly black coat and its F_1 progeny from its mating to an ICR female mouse.

(E) A 16-week-old gPSC-S2 4N-comp mouse with a uniformly black coat and its F_1 progeny from its mating to an ICR female mouse.

(F) The global DNA methylation levels of SSCs (p20), gPSCs (p25), 5C-gPSCs (p25), and ESCs (p10) around the CGI regions and gene bodies of indicated samples. Samples are indicated by colors.

Figure S4. Sample information and quality control of single cell RNA-sequencing data of 5C induced SSC reprogramming, related to Figure 4

(A) Sample information of DMSO or 5C induced reprogramming cells for scTrio-seq2 (transcriptome) analysis in this study.

(B) Boxplot showing the numbers of genes (top) and UMIs (bottom) detected in each single cell collected at different time points, corresponding to Figure S4A.

(C) UMAP plots showing the integration of transcriptome data from the original condition (control), DMSO and 5C induced reprogramming cells, cells are colored by clusters (left and middle) and batches (right).

(D) UMAP plot showing the integration of transcriptome data from the successful branch of SSC reprogramming (DMSO and 5C groups), mouse *in vivo* germ cells and gonadal somatic cells from E6.5 to PND5.5. Orange dashed arrow indicates the trajectory of *in vivo* germ cell development, red dashed arrow indicates the trajectory of SSC reprogramming. Cells are colored by batches.

(E) UMAP plots showing the integration of transcriptome data from the successful branch of SSC reprogramming (control group), mouse *in vivo* germ cells and gonadal somatic cells from E6.5 to PND5.5. Grey dots indicate *in vivo* germ cells or gonadal somatic cells, colored dots with black outline indicate reprogramming cells or ESCs (left). Cells are colored by clusters (left) and batches (right). Orange dashed arrow indicates the trajectory of *in vivo* germ cell development, green dashed arrow indicates the trajectory of SSC reprogramming.

(F) Scmap projection of the successful branch between SSC reprogramming (control group) and mouse *in vivo* germ cells. The color key from bright yellow to dark blue indicates low to high ratio of cells projected.

(G) Violin plots showing the expression levels of stage-specific genes during *in vivo* germ cell development.

(H) Immunofluorescence of *Oct4*-EGFP (green) co-stained with DPPA3 (pink) and TFAP2C (red) in domed colonies during SSC reprogramming. Scale bar, 50 μm.

Figure S5. Identification of gene editing in SSC reprogramming, related to Figure 5

(A) SCENIC TF-targets network of representative regulons highlighted in Figure 5B, target genes related GO terms in Figure 5B were labeled.

(B) q-PCR analysis of the relative expression of *Rhox5*, *Otx2*, and *Prdm14* normalized to the geometric mean of *Rps2* and *Gapdh* of CRISPR-CasRx transfected gPSCs. Unpaired two-tailed Student's *t*-test, $*P < 0.05$, $**P < 0.01$.

(C) Relative expression of RHOX5 (top), PRDM14 (middle), and OTX2 (bottom) measured by western blotting of sgNC-, sg*Rhox5*/*Prdm14*/*Otx2*-transfected gPSCs.

(D) Bright field images of SSCs transfected with CRISPR-CasRx for targeting empty vector and *Rhox5*, respectively. Scale bar, 200 μm.

(E-G) Immunofluorescence of ZBTB16 (top, red) co-stained with OCT4 (top, green), GFRA1 (bottom, red) co-stained with DDX4 (bottom, pink) in sg*Rhox5* 2#-, sg*Prdm14* 2#-, and sg*Otx2* 2#-transfected SSCs. Scale bars, 20 μm.

(H) Bar plots showing the number of OG(+) colonies reprogrammed from sgNC-, sg*Prdm14*-, and sg*Otx2* transfected SSCs in 5C condition on day 19. Unpaired two-tailed Student's *t*-test, $**P < 0.01$.

(I) (Top) Immunofluorescence of *Oct4*-EGFP (green) co-stained with RHOX5 (i, red) and DPPA3 (ii, pink) in OG(+) colonies reprogrammed from sgNC- and sg*Rhox5* 2#-transfected SSCs. Scale bars, 50 μm; (bottom) Dot plots showing the relative intensity of RHOX5 and DPPA3 in OG(+) colonies corresponding to (i) and (ii), respectively. Unpaired two-tailed Student's *t*-test, $****P < 0.0001$.

Figure S6. Quality control of single cell methylome data and methylation signatures at specific elements and regions during SSC reprogramming, related to Figure 6

(A) Sample information of DMSO or 5C induced reprogramming cells for scTrio-seq2 (DNA methylome) analysis in this study.

(B) Boxplots showing the numbers of CpG, 1X CpG depth, mapping ratio and bisulfite conversion rate (BSCR) detected in DMSO (top)- and 5C (bottom)-induced reprogramming cells.

(C) The DNA methylation level around the CGI regions (top) and gene bodies (bottom) of all genes in DMSO and 5C induced reprogramming cells.

(D) Volcano plots showing the correlation between gene expression and the DNA methylation level of the corresponding promoters in the successful branch of SSC reprogramming. The *x* axis represents the differential expression (genes with $|\log(\text{fold change})| \geq 1$, adjusted $P < 0.01$ were designated as differential expressed genes, DEGs) between comparison groups, and positive values indicate the higher expression in RPG2/3/4. The *y* axis represents the DNA methylation difference of promoter (genes with absolute methylation difference > 30% and $P < 0.05$ were designated as differential methylated promoter genes, DMPGs) between the comparison groups, and negative values indicate the lower methylation in RPG2/3/4. Red dots indicate the DEGs with differentially methylated promoters (DEGs & DMPGs). Blue dots indicate the DEGs without differentially methylated promoters (only DEGs).

(E) Pearson correlation coefficient between gene expression and promoter DNA methylation levels during SSC reprogramming process of DEGs & DMPGs in (Figure S6D) and genes in Figure 6B (n = 119). Histogram in the top showing the distribution of genes. Genes were ordered by Pearson correlation coefficient.

(F) Multidimensional scaling (MDS) analysis on the DNA methylome data of SSC reprogramming cells, SSCs, mouse *in vivo* germ cells and sperm, corresponding to Figure 6C. Clusters are indicated by colors.

(G) Unsupervised hierarchical clustering based on the DNA methylome data of SSC reprogramming cells, SSC, mouse *in vivo* germ cells and sperm.

(H) Boxplots showing the DNA methylation dynamics of indicated genomic features during DMSO and 5C induced reprogramming processes, respectively.

(I) Boxplots showing the DNA methylation dynamics of representative maternal ICRs during DMSO and 5C induced reprogramming processes, respectively. The DNA methylation level of maternal ICRs in sperm was the reference for other samples.

Figure S7. DNA methylation dynamics at specific elements and regions during the late stage of SSC reprogramming, related to Figure 7

(A) Heatmap showing the Pearson's correlation between RPG1/2/3/4 and gPSCs (p1 and p15) based on transcriptome data.

(B) Unsupervised hierarchical clustering of ESCs, RPG1/2/3/4, and different passages of gPSCs and 5C-gPSCs based on DNA methylome data.

(C) The DNA methylation level around the CGI regions and gene bodies of all genes in gPSCs, 5C-gPSCs, and CiPSCs.

(D) Three-dimensional MDS analysis of SSCs, ESCs, gPSCs, 5C-gPSCs and CiPSCs based on DNA methylome data. Clusters are colored by samples.

(E) Boxplots showing the DNA methylation level of the indicated genomic features in SSCs, ESCs, different passages of gPSCs, 5C-gPSCs and CiPSCs.

(F) Boxplots showing the DNA methylation level of representative paternal ICRs in sperm, SSCs, ESCs, different passages of gPSCs and 5C-gPSCs.

(G) Lollipop methylation diagrams showing the DNA methylation level of representative maternal ICR (*Peg3*) and paternal ICR (*H19*) in SSCs, ESCs, p25 gPSCs and 5C-gPSCs by sequencing reads in single CpG site resolution.

(H) E12.5 embryos (left) produced by tetraploid complementation (4N-comp) of two CiPSC lines, respectively, and their corresponding E12.5 genital ridges (middle and right). Scale bar, 2 mm (left), 500 µm (middle and right).

(I) Boxplots showing the DNA methylation level of representative maternal and paternal ICRs in ESCs and different passages of CiPSCs.

(J) Line plot showing the mean methylation levels of *Dlk1-Dio3* region in ESCs (p10), R-1 CiPSCs (p5 and p25), K-2 CiPSCs (p5 and p25), respectively. Grey shadows indicate gene regions, the red shadow indicates IG-*Gtl2* ICR region.

(K) Box plots showing the mean DNA methylation levels across gene bodies (from transcription start site (TSS) to transcription end site (TES)) and the 50-bp flanking regions of each miRNA encoded locus in *Dlk1-Dio3* region in ESCs and CiPSCs. One dot represents a miRNA encoded locus, the same loci in distinct PSCs are connected by grey lines. Paired two-tailed Student's *t*-test was performed between ESCs and CiPSCs.

Figure S1

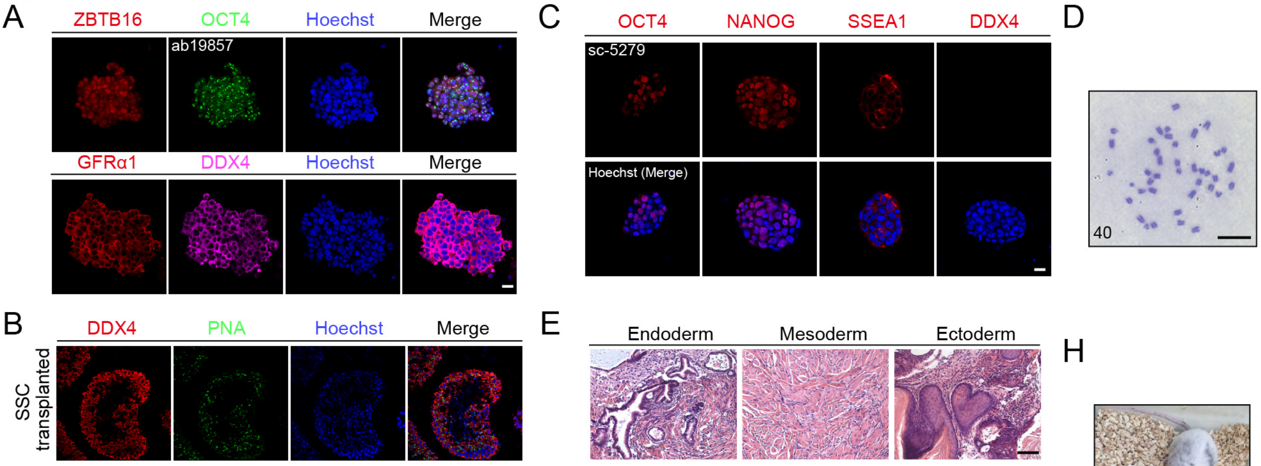

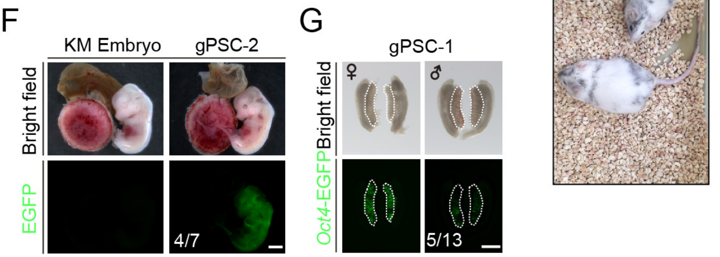

I

Cell Line	Cell Line Passages	No. Injected Embryos	No. Recipient Mice	No. Development *in vivo* (%)		
				Full-Term	Live Adult	Chimera
gPSC-1	p10, p12	40	4	4	4	2/4 (50)

J

Cell type	Day 0	Day 8	Day 10	Day 12	Day 14	Day 16	Day 18	Day 20	Day 23	Day 27	gPSC-1 p1	gPSC-1 p15	F1 4-5 mESC
Cell number	95	87	93	283	566	571	656	846	669	473	48	32	45

Total After QC : 4,464

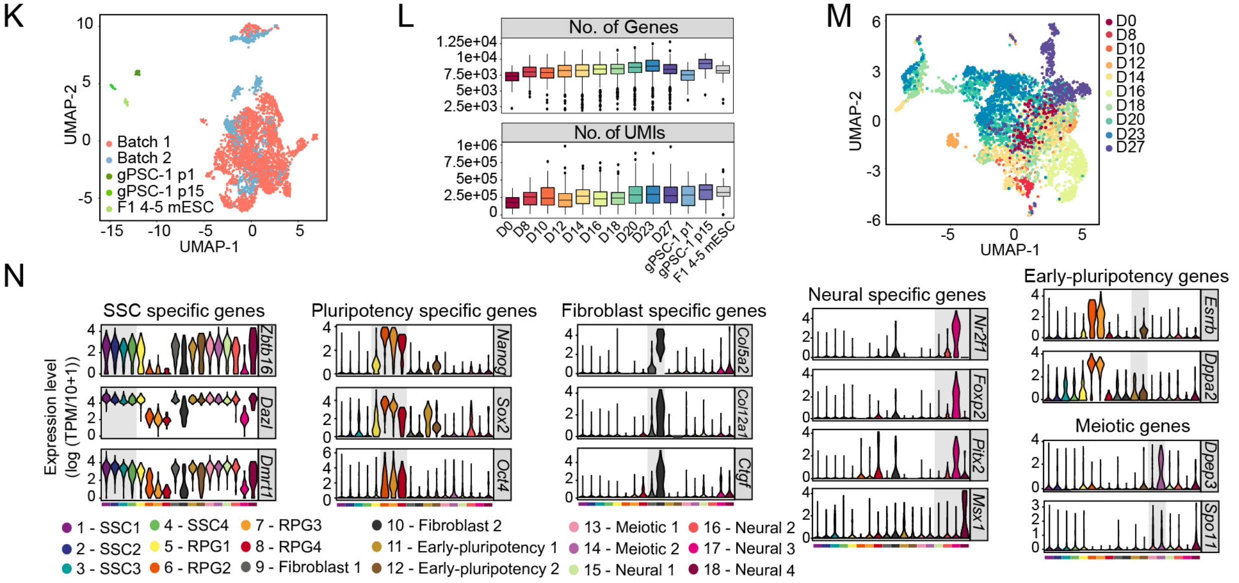

Figure S2

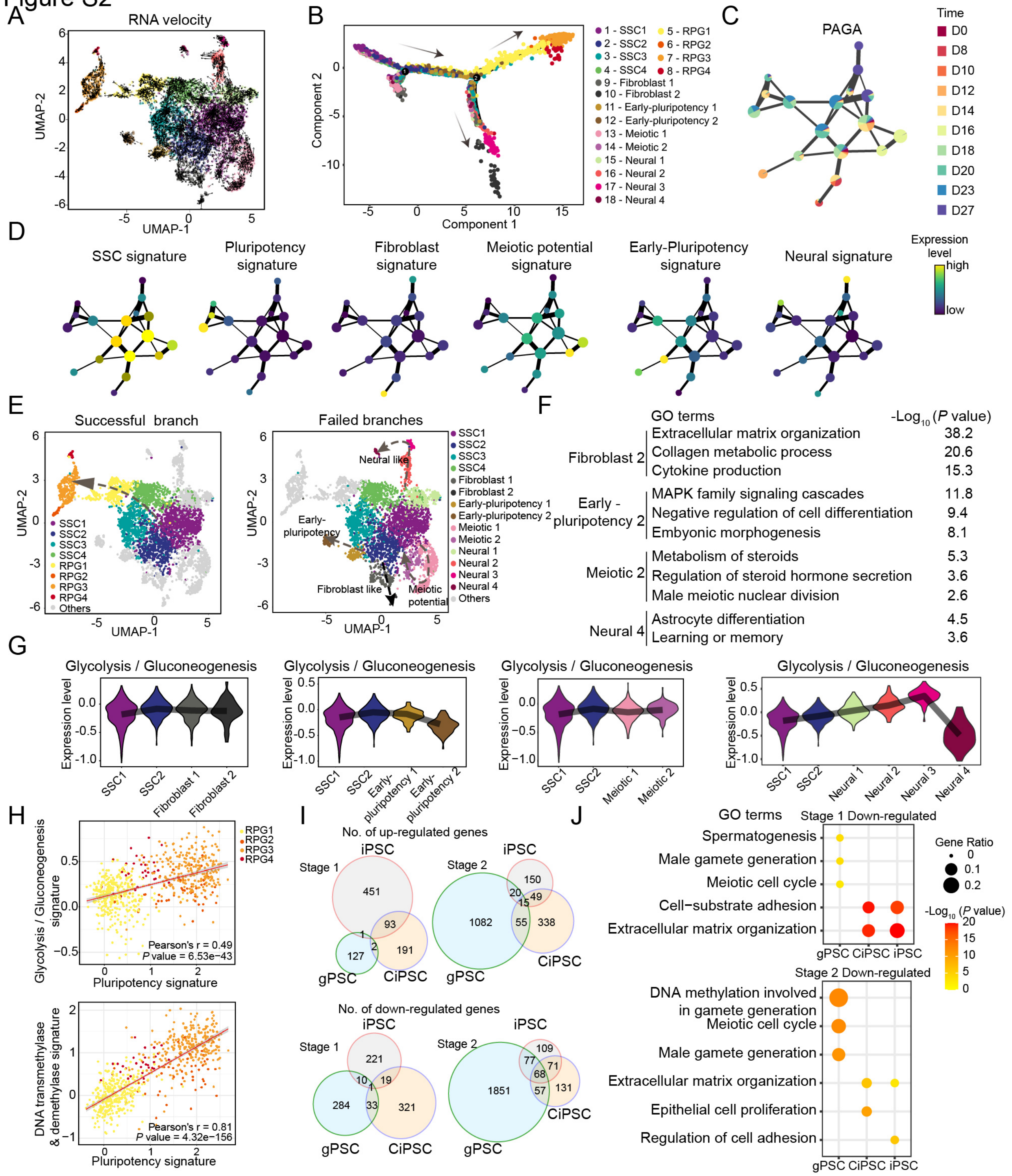
A
RNA velocity
UMAP-1
UMAP-2
B
Component 1
Component 2
1 - SSC1
2 - SSC2
3 - SSC3
4 - SSC4
5 - RPG1
6 - RPG2
7 - RPG3
8 - RPG4
9 - Fibroblast 1
10 - Fibroblast 2
11 - Early-pluripotency 1
12 - Early-pluripotency 2
13 - Meiotic 1
14 - Meiotic 2
15 - Neural 1
16 - Neural 2
17 - Neural 3
18 - Neural 4
C
PAGA
Time
D0
D8
D10
D12
D14
D16
D18
D20
D23
D27
D
SSC signature
Pluripotency signature
Fibroblast signature
Meiotic potential signature
Early-Pluripotency signature
Neural signature
Expression level
high
low
E
Successful branch
Failed branches
Neural like
Early-pluripotency
Fibroblast like
Meiotic potential
Others
F
GO terms
-Log10 (P value)
Fibroblast 2
Extracellular matrix organization 38.2
Collagen metabolic process 20.6
Cytokine production 15.3
Early - pluripotency 2
MAPK family signaling cascades 11.8
Negative regulation of cell differentiation 9.4
Embyonic morphogenesis 8.1
Meiotic 2
Metabolism of steroids 5.3
Regulation of steroid hormone secretion 3.6
Male meiotic nuclear division 2.6
Neural 4
Astrocyte differentiation 4.5
Learning or memory 3.6
G
Glycolysis / Gluconeogenesis
Expression level
H
Glycolysis / Gluconeogenesis signature
Pluripotency signature
Pearson's r = 0.49
P value = 6.53e−43
DNA transmethylase & demethylase signature
Pearson's r = 0.81
P value = 4.32e−156
I
No. of up-regulated genes
No. of down-regulated genes
Stage 1
Stage 2
iPSC
gPSC
CiPSC
J
GO terms
Stage 1 Down-regulated
Spermatogenesis
Male gamete generation
Meiotic cell cycle
Cell−substrate adhesion
Extracellular matrix organization
Stage 2 Down-regulated
DNA methylation involved in gamete generation
Epithelial cell proliferation
Regulation of cell adhesion
Gene Ratio
-Log10 (P value)

Figure S3

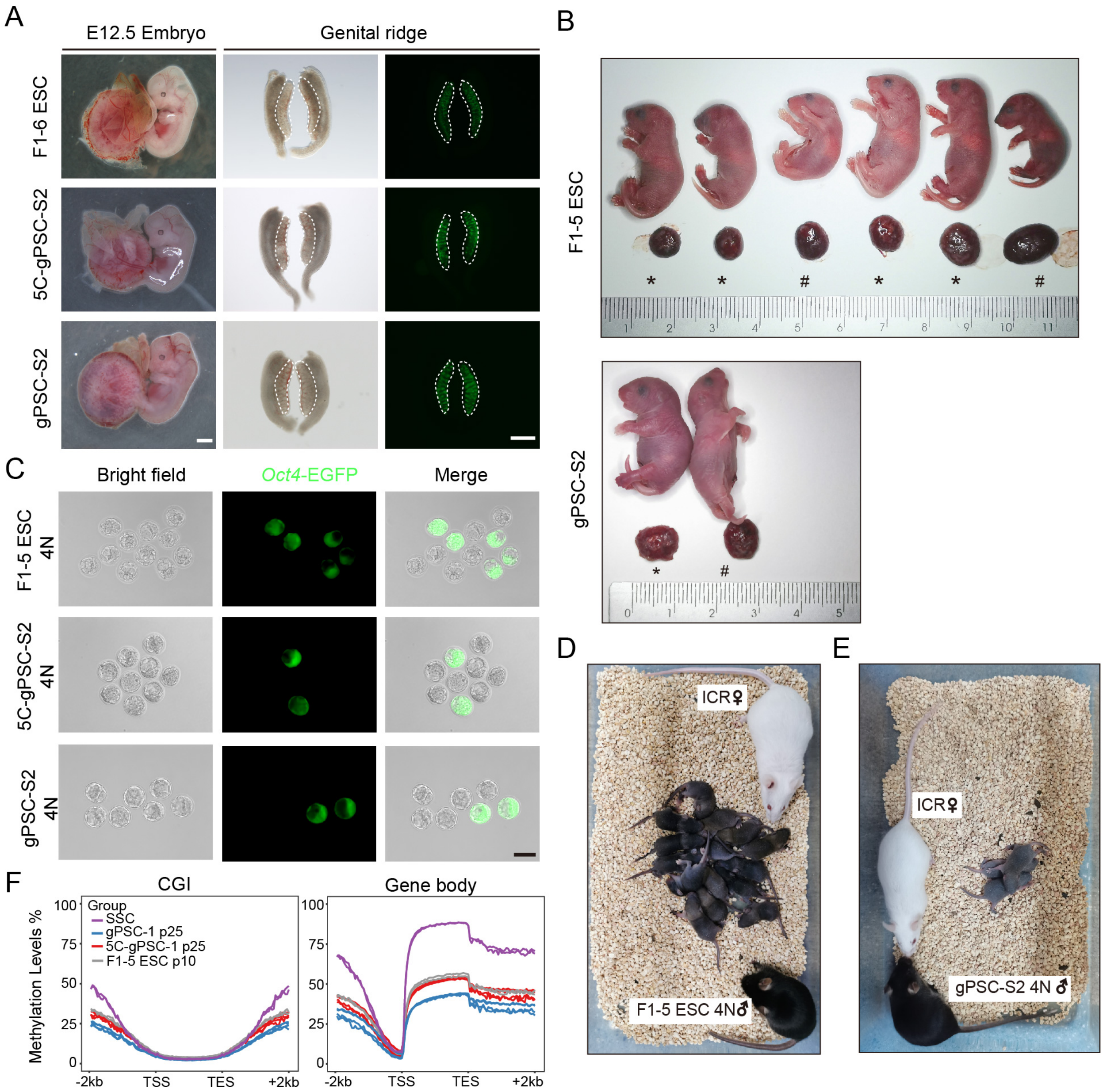
A
E12.5 Embryo
Genital ridge
F1-6 ESC
5C-gPSC-S2
gPSC-S2
B
F1-5 ESC
gPSC-S2
C
Bright field
Oct4-EGFP
Merge
F1-5 ESC 4N
5C-gPSC-S2 4N
gPSC-S2 4N
D
ICR♀
F1-5 ESC 4N♂
E
ICR♀
gPSC-S2 4N ♂
F
CGI
Gene body
Methylation Levels %
Group
SSC
gPSC-1 p25
5C-gPSC-1 p25
F1-5 ESC p10
-2kb
TSS
TES
+2kb

Figure S4

A

DMSO	Day 0	Day 20	Day 24
Cell number	8	266	91

5C	Day 12	Day 14	Day 16
Cell number	368	180	176

Total After QC : 1,089

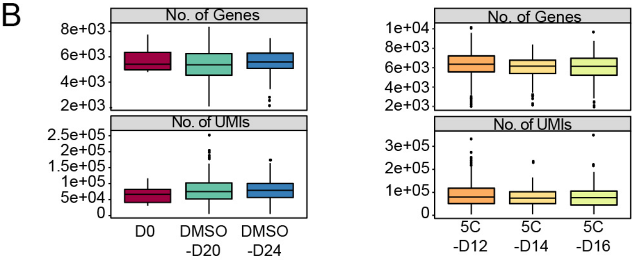

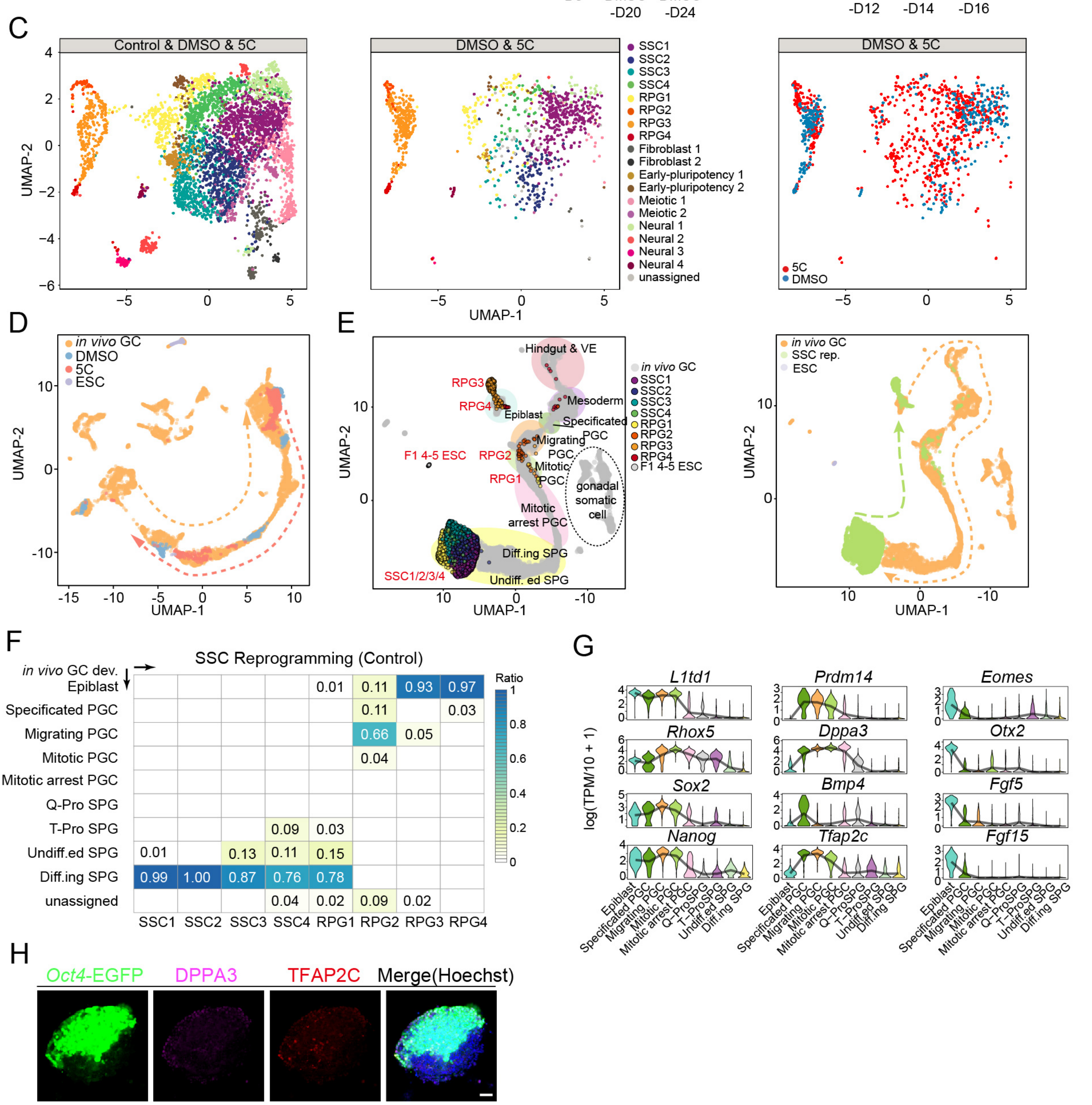

Figure S5

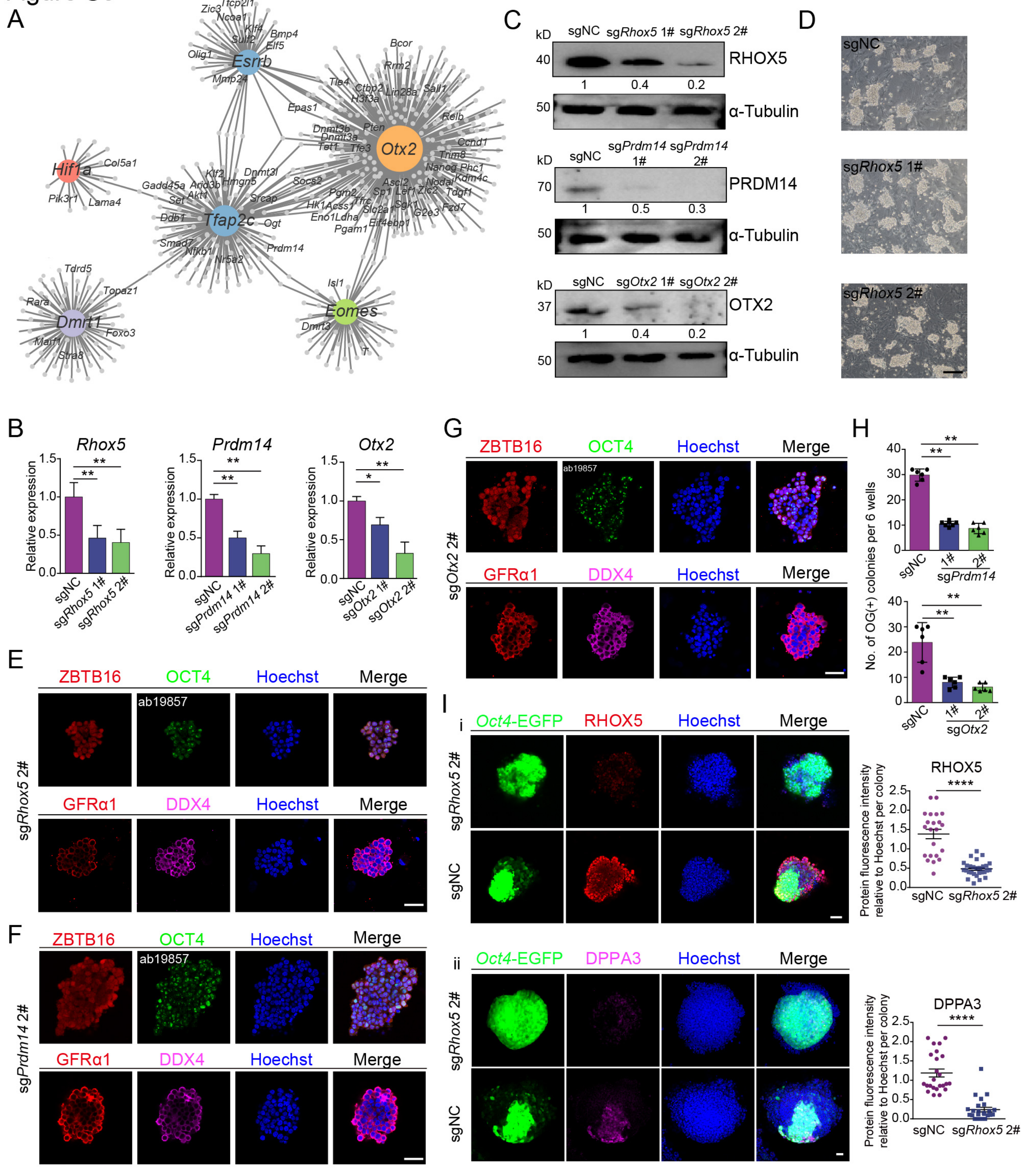
A
Esrrb
Otx2
Hif1a
Tfap2c
Dmrt1
Eomes
C
kD
sgNC
sgRhox5 1#
sgRhox5 2#
RHOX5
α-Tubulin
PRDM14
OTX2
D
B
Rhox5
Prdm14
Otx2
Relative expression
E
ZBTB16
OCT4
Hoechst
Merge
GFRα1
DDX4
F
G
H
No. of OG(+) colonies per 6 wells
I
Oct4-EGFP
RHOX5
DPPA3
Protein fluorescence intensity relative to Hoechst per colony

Figure S6

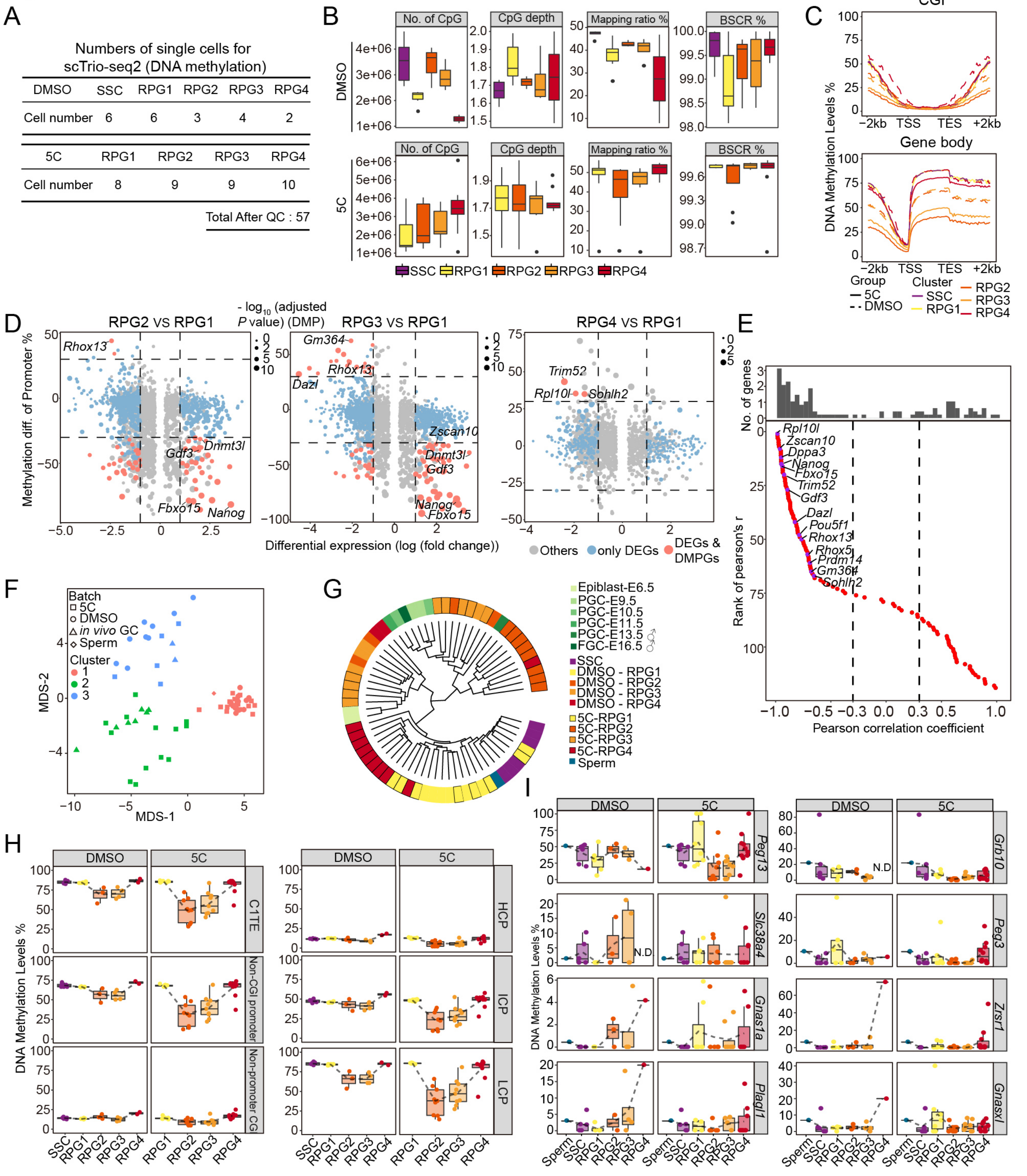

A
Numbers of single cells for scTrio-seq2 (DNA methylation)
DMSO SSC RPG1 RPG2 RPG3 RPG4
Cell number 6 6 3 4 2
5C RPG1 RPG2 RPG3 RPG4
Cell number 8 9 9 10
Total After QC : 57
B
No. of CpG
CpG depth
Mapping ratio %
BSCR %
DMSO
5C
SSC RPG1 RPG2 RPG3 RPG4
C
CGI
Gene body
DNA Methylation Levels %
−2kb TSS TES +2kb
Group
5C
DMSO
Cluster
SSC
RPG1
RPG2
RPG3
RPG4
D
RPG2 VS RPG1
RPG3 VS RPG1
RPG4 VS RPG1
- log10 (adjusted P value) (DMP)
Methylation diff. of Promoter %
Differential expression (log (fold change))
Others
only DEGs
DEGs & DMPGs
Rhox13
Gdf3
Dnmt3l
Fbxo15
Nanog
Gm364
Dazl
Zscan10
Trim52
Rpl10l
Sohlh2
E
No. of genes
Rank of pearson's r
Pearson correlation coefficient
Rpl10l
Zscan10
Dppa3
Nanog
Fbxo15
Trim52
Gdf3
Dazl
Pou5f1
Rhox13
Rhox5
Prdm14
Gm364
Sohlh2
F
Batch
5C
DMSO
in vivo GC
Sperm
Cluster
1
2
3
MDS-1
MDS-2
G
Epiblast-E6.5
PGC-E9.5
PGC-E10.5
PGC-E11.5
PGC-E13.5 ♂
FGC-E16.5 ♂
SSC
DMSO - RPG1
DMSO - RPG2
DMSO - RPG3
DMSO - RPG4
5C-RPG1
5C-RPG2
5C-RPG3
5C-RPG4
Sperm
H
DMSO
5C
C1TE
Non-CGI promoter
Non-promoter CG
HCP
ICP
LCP
DNA Methylation Levels %
SSC RPG1 RPG2 RPG3 RPG4
I
DMSO
5C
Peg13
Slc38a4
Gnas1a
Plagl1
Grb10
Peg3
Zrsr1
Gnasxl
N.D
DNA Methylation Levels %
Sperm SSC RPG1 RPG2 RPG3 RPG4

Figure S7

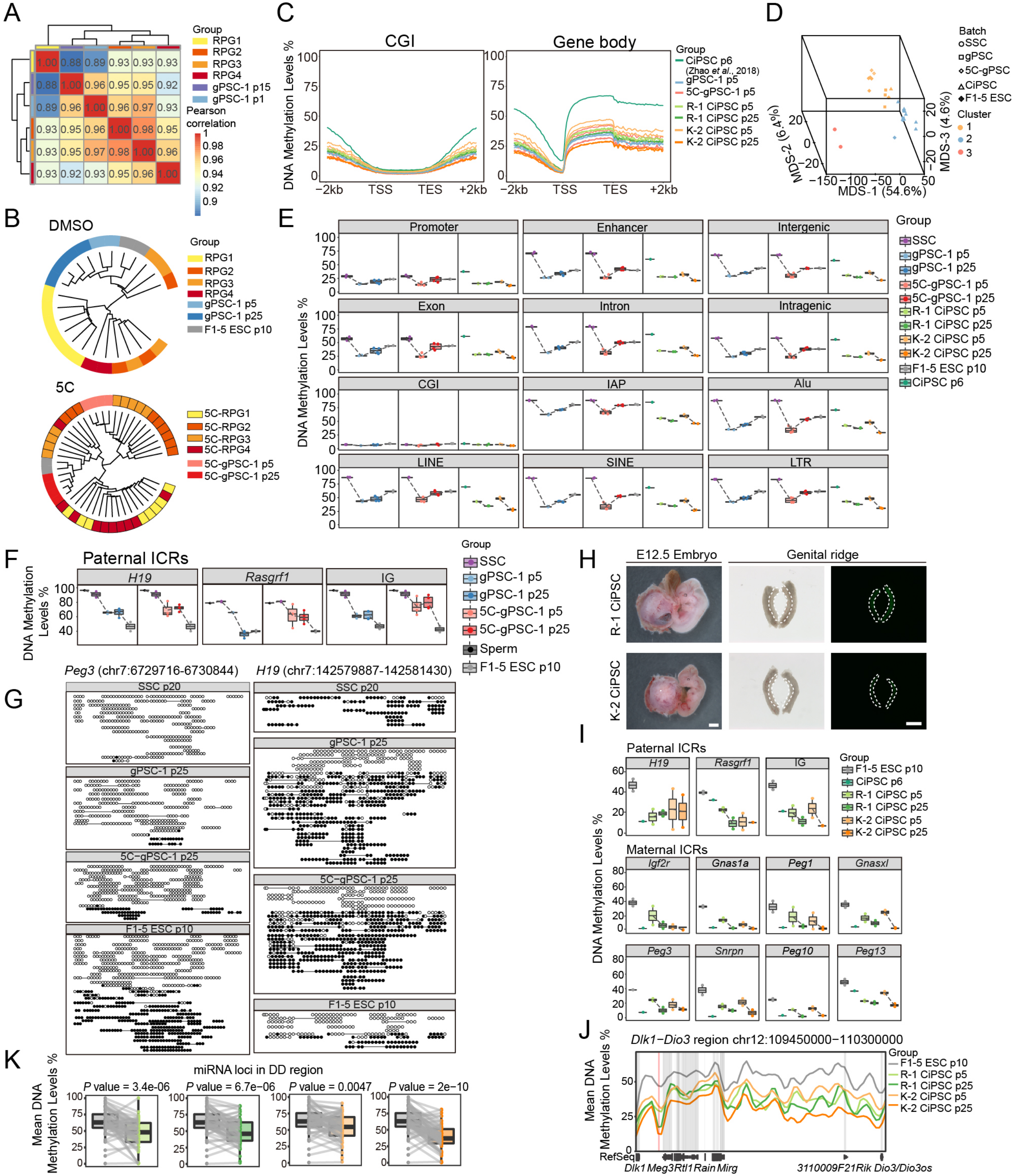

A
B
C
D
E
F
G
H
I
J
K
Group
Pearson correlation
DMSO
5C
CGI
Gene body
DNA Methylation Levels %
Batch
Cluster
MDS-1 (54.6%)
MDS-2 (6.4%)
MDS-3 (4.6%)
Promoter
Enhancer
Intergenic
Exon
Intron
Intragenic
IAP
Alu
LINE
SINE
LTR
Paternal ICRs
Maternal ICRs
Peg3 (chr7:6729716-6730844)
H19 (chr7:142579887-142581430)
E12.5 Embryo
Genital ridge
R-1 CiPSC
K-2 CiPSC
Dlk1−Dio3 region chr12:109450000−110300000
miRNA loci in DD region
Mean DNA Methylation Levels %

Table S1. Sample Information of 4,464, 1,089, 57 Cells for scSTRT-seq and scTrio-seq2 (transcriptome), scTrio-seq2 (DNA methylation), respectively, related to Figures 1C and 6A, Figures S1J, S1K, S4A, S4C, and S6A.

Table S2. DEGs and GO Terms of Four Clusters in the Successful and Failed Reprogramming Branches, respectively, related to Figure 1H and Figure S2F. Genes sets used to calculate gene signatures in Figure S2H.

Table S3. DEGs and GO Terms of Two Stages amongst SSC Reprogramming and Two MEF-reprogramming Processes, related to Figures 1M and 1N, Figures S2I and S2J.

Table S4. Candidate compounds for chemical screening, Primers for SSLP, Genotyping and q-PCR. Guide RNAs for Gene Knockdown, related to Figures 2A, 3B, 3C, 3O, and 5D, Figure S5B.

Table S5. GO Terms of 836 Stage-specific Genes of *in vivo* Germ Cell Expressed in the Corresponding Clusters of SSC Reprogramming, related to Figure 4C.

Table S6. DEGs and SCENIC Analysis of Each Cluster in the Successful Reprogramming Branch, related to Figures 5A, 5B and 5C, Figure S5A.

Table S7. DEGs and DMPGs of RPG1/2/3/4, related to Figure S6D and S6E.

Table S8. GO Terms of Up-regulated Genes of RPG2 and RPG3 from 5C Group than that from Control and DMSO Groups, related to Figure 6H.
